# Model based on COVID-19 evidence to predict and improve pandemic control

**DOI:** 10.1371/journal.pone.0286747

**Published:** 2023-06-15

**Authors:** Rafael I. González, Pablo S. Moya, Eduardo M. Bringa, Gonzalo Bacigalupe, Muriel Ramírez-Santana, Miguel Kiwi

**Affiliations:** 1 Centro de Nanotecnología Aplicada, Universidad Mayor, Santiago, Chile; 2 Center for the Development of Nanoscience and Nanotechnology, CEDENNA, Santiago, Chile; 3 Departamento de Física, Facultad de Ciencias, Universidad de Chile, Santiago, Chile; 4 CONICET, Facultad de Ingeniería, Universidad de Mendoza, Mendoza, Argentina; 5 School of Education and Human Development, University of Massachusetts Boston, Boston, MA, United States of America; 6 CreaSur, Universidad de Concepción, Concepción, Chile; 7 Departamento de Salud Pública, Facultad de Medicina, Universidad Católica del Norte, Coquimbo, Chile; Texas Christian University, UNITED STATES

## Abstract

Based on the extensive data accumulated during the COVID-19 pandemic, we put forward simple to implement indicators, that should alert authorities and provide early warnings of an impending sanitary crisis. In fact, Testing, Tracing, and Isolation (TTI) in conjunction with disciplined social distancing and vaccination were expected to achieve negligible COVID-19 contagion levels; however, they proved to be insufficient, and their implementation has led to controversial social, economic and ethical challenges. This paper focuses on the development of simple indicators, based on the experience gained by COVID-19 data, which provide a sort of yellow light as to when an epidemic might expand, despite some short term decrements. We show that if case *growth* is not stopped during the 7 to 14 days after onset, the growth risk increases considerably, and warrants immediate attention. Our model examines not only the COVID contagion propagation speed, but also how it accelerates as a function of time. We identify trends that emerge under the various policies that were applied, as well as their differences among countries. The data for all countries was obtained from *ourworldindata.org*. Our main conclusion is that if the reduction spread is lost during one, or at most two weeks, urgent measures should be implemented to avoid scenarios in which the epidemic gains strong impetus.

## Introduction

The COVID-19 pandemic has dramatically affected all of humanity, but it also provided an enormous amount of data which may provide valuable lessons on how to effectively face future events of similar characteristics. Already a wealth of statistical data analysis has been published, and models to predict epidemic evolution have been developed. A few recent examples are the work of Biggerstaff *et al.* [1] who obtained from early insights values for the range of incubation period and doubling time, and observed that due to sanitary interventions the effective reproductive number varied significantly. Brizzi *et al.* [2] pointed out that there is a tendency to overestimate the parameter *R*_0_ and the basic effective reproduction *R*_*t*_ during the onset of an epidemic. Gopalakrishnan *et al.* pointed out that data otherwise reliable obtained for a specific geographical region, no matter how large, does not necessarily apply in other locations [3]. Moreover, they stress that state-level data are defective, when applied to policy decisions for smaller areas, since they lead to errors which can be as large as 200 to 300%.

McCabe *et al.* [4] mathematical transmission models, while widely used, are pieces of evidence that have to be applied carefully, since they are uncertain and cannot be adopted uncritically to develop public policy interventions. These conclusions are moderated by the development of increasingly complex data treatments, but they always should be critically examined. Later on [5] they focused their interest on Great Britain, but expanded its scope to develop a more complete understanding of the complex relationships between models, decision-making, the media and the public. Their interest was to document the history of COVID-19, and draw lessons to improve response when future pandemic outbursts occur. They also stressed scientists responsibility to ensure that their models are of the highest scientific quality, but at the same time acknowledge and point out their limitations.

Much along the same lines Poletto, Scarpino and Volz [6] observed that in spite of tremendous advances, predictions of epidemic evolution are prone to errors, and justify their assertion indicating that to make their use reliable the variables to be estimated have to be clearly defined, and the people who use the predictions must have a full understanding of their limitations. Similarly, Brooks-Pollock *et al.* [7] discuss how modelling can help to develop adequate policy making. In addition, they also examine pitfalls of publishing practices and academic credit and stress the importance of transparency and reproducibility. Brauner *et al.* [8] examined government interventions during the first COVID-19 wave. They did so in 41 different countries and observed that the effect varied widely from one country to another, but provided insights into which interventions could help to maintain social and economic activities during the pandemic. Sonabend *et al.* [9] studied the requirements and duration for the lifting of non-pharmaceutical interventions (NPI), and developed a mathematical model to examine the process. On this basis they predicted the onset of a new COVID wave, which actually materialized.

Parag *et al.* [10] focused on early epidemic warning signals, or when a new wave looms, to put forward an early warning approach to reduce as much as feasible its negative impacts. Their strategy is to employ, as far as possible, the information extracted from low-incidence periods data, which is by nature scarce, in order to better handle a forthcoming event. In fact our approach, which uses a completely different methodology, is quite analogous in terms of objectives. We make use of the vast amount of data that is now available in order to extract information that could be useful in similar future event. Detailed analysis of the policies used in different countries are now available [11]. However, challenges to estimate the threat posed by the pandemic in order to plan effective interventions [12, 13], that are specific and have precise timing [14, 15], remain an open challenge.

There are many NPIs, but reducing human interactions [15], as well as reducing superspreading events [16] and to consider the relevance of airborne transmission of the virus [17, 18], have proven to be crucial. In addition, also powerful pharmaceutical resources became available, in particular the development of the highly effective drug Paxlovid played a major role. The Test, Trace, and Isolate (TTI) strategy has been shown to contribute to decrease epidemic growth [11, 19], when the number of new cases is low and they are feasible to trace. Otherwise, confinement is a control alternative when the number of new cases is threatening to overload health services, but has an enormous social and economic cost [20]. The NPI aims at reducing the number of infections and/or reaching the “ideal” of zero infected cases. Available data shows that a type of NPI that succeeds in one country might not be effective in another. Quite often, the data are unreliable [21, 22] due to different methodologies used to collect and process the data, among other reasons (asymptomatic cases, lack of testing, and false-negative RT-PCR tests) [23–28]. But, in spite of the latter, actions are needed. With this purpose, we put forward a model focused on identifying the early risk signals that the available data reveal. To do so, we consider, in addition to the number of cases and their time evolution, how the increase or decrease of cases varies with time. In other words, in close analogy to the dynamics of motion, we incorporate in our model the speed of change of the number of infections. Ironically, we use tools developed by Isaac Newton during the great plague epidemic that occurred in England during 1665–1666.

At this point, it is essential to clarify our approach. Having zero contagion should not be taken literally; COVID-19 will likely continue to circulate for a long time [29]. However, while there is no fully effective treatment, or while massive and equitable access to immunization with an effective vaccine, and novel treatments, are not universally available [30, 31] it is reasonable to aim at contagion reduction. Therefore, there is a lesson that can be applied to future pandemics: do your best to eliminate contagion while science works on solutions that prevent the epidemic spread, illness, and death.

To control the pandemic in 2020 the WHO recommended to achieve “…a decline of infections of at least 50% over a 3-week period since the latest peak, and a continuous decline in the observed incidence of confirmed and probable cases…” [32]. This is sound advice from a global health perspective, but difficult to implement. Our paper intends to contribute to the development of a set of indicators that provide quantitative criteria in line with the WHO recommendation, and therefore, should be helpful parameters for decision and policy makers.

Based on the extensive data accumulated during the COVID-19 pandemic we put forward simple to implement indicators, that should alert authorities and provide early warnings of an impending sanitary crisis.

## Methods of data analysis

**Fig 1:**
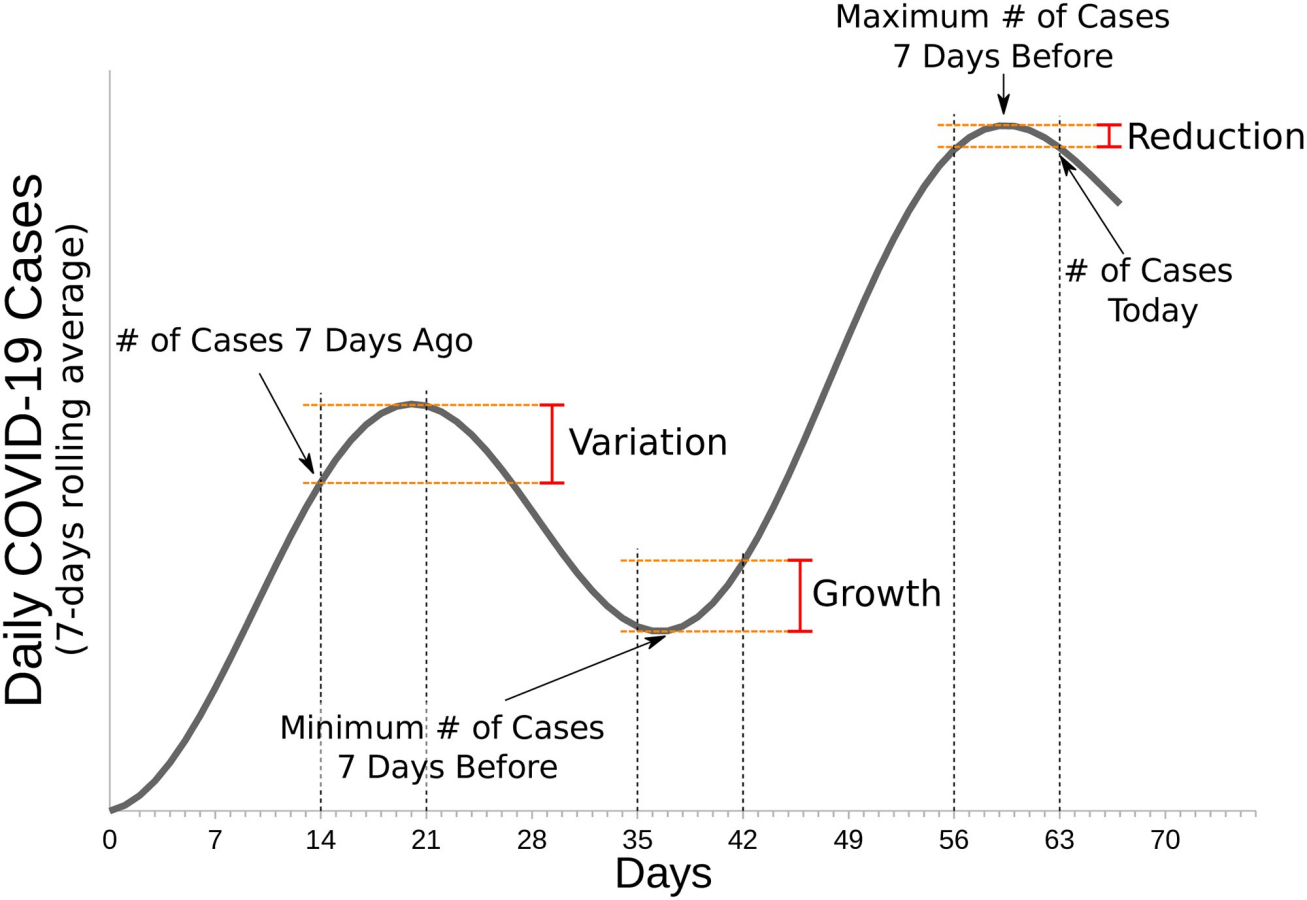
Diagram to illustrate how the proposed performance variables are defined and calculated. The variables are the reduction *R*_*d*_, the growth *G*, and the variation rate *V*.

We start defining the seven-days reduction, illustrated in Fig 1, as

$$\begin{array}{c} R_{d}\left(t\right)=\frac{N\left(t\right)-N_{max}}{N(t)} \end{array}$$ (1)

where *t* labels a specific day, *N*(*t*) is the 7-days rolling average number of cases from date *t* − 6 to *t* (both included), and *N*_*max*_ corresponds to the maximum of *N*(*t*) during the previous seven days, *i.e.*, between days *t* − 7 and *t* − 1. This way, when the average number of infections decreases *R*_*d*_ < 0.

The growth *G* instead is the weekly increase relative to the minimum average number of infections *N*_*min*_, that is

$$\begin{array}{c} G\left(t\right)=\frac{N\left(t\right)-N_{min}}{N_{min}} \end{array}$$ (2)

where *N*_*min*_ corresponds to the minimum *N*(*t*) during the previous seven days (from date *t* − 7 to *t* − 1). The variation *V* is usually defined as follows:

$$\begin{array}{c} V\left(t\right)=\frac{N(t)-N(t-7)}{N(t-7)}\; \end{array}$$ (3)

The advantage of defining *R*_*d*_(*t*) and *G*(*t*) before *V*(*t*) is that the first two can be symmetric. As a simple example, if we have an average linear increment of cases with one case at day 0, 8 at day 7, and 1 at day 14, we obtain *G*(7) = 7, $G\left(14\right)=-\frac{1}{2}$, $R_{d}\left(7\right)=\frac{1}{8}$, *R*_*d*_(14) = −7, *V*(7) = 7, $V\left(14\right)=-\frac{7}{8}$. We observe that *G*(7) = −*R*_*d*_(14), but the variation is not symmetric. We propose to use *G*(*t*) and *R*_*d*_(*t*), instead of the usual *V*(*t*) because, in the best-case scenario, we can observe symmetric growth and reduction. In practice, we generally observed a larger growth of cases rather than their reduction. With this in mind, we stress the importance of maintaining the case reduction for as long as possible.

Finally, all these time variations can be associated with a corresponding “velocity.” We define the growth velocity as

$$\begin{array}{c} v_{G}\left(t\right)=\frac{dG(t)}{dt} \end{array}$$ (4)

where the derivative is computed numerically using finite differences.

The data for all countries were obtained from *ourworldindata.org* [33] on January 17, 2022, including deaths and population data. As in the early stages of the epidemic the data does vary a lot, all the calculations of variations were made once a country reported more than 100 accumulated cases. Similarly, considering that the data can vary significantly from day to day, we calculate the seven days moving centered case average [34]. The rest of the variation averages are obtained as the rolling seven day average.

The reduction, growth, and variation were computed from the average daily cases. We define the daily changes of the variation, named as velocity, by means of finite differences. For example, the reduction velocity is computed as follows:

$$\begin{array}{ccc} v_{R}\left(t\right) & = & \frac{dR_{d}\left(t\right)}{dt}\\ & \approx & \frac{R_{d}\left(t-2\right)-8R_{d}\left(t-1\right)+8R_{d}\left(t+1\right)-R_{d}\left(t+2\right)}{12} \end{array}$$ (5)

where *R*_*d*_ is the reduction and *t* is the date. In this case, we use a 4-day central finite difference.

During the first weeks of the epidemic, the velocity data can be quite noisy, so we decided to average the velocity reduction, growth, or variation (as appropriate) during the first three weeks once 100 cases have been reached. During the rest of the days, it is calculated directly on the variations without averaging. We present the averaged speed every seven days in the curves, so that they look smoother.

To calculate the total variation of Fig 5 we added all the daily reductions without averaging, and they were divided by the total number of days since the first 100 accumulated cases were exceeded. The reduction was multiplied by 100 and expressed as a positive number for clarity.

Finally, it is worth mentioning that for six countries (China, France, Uganda, Mexico, Spain, and United Kingdom) we found some anomalous data that caused the calculation of the variations to be altered (details are given in the S1 File). This is because these countries made data corrections during the emergency. As the spirit of this work is to keep it simple, we decided to replace those values with the average number of cases during the previous 7 days. For France this had to be done several times, and some were less than 7 days apart. Therefore, this procedure had to be implemented repeatedly in time. Some special days for France were October 25 and 26, as well as between November 2 and 4, and what we did was to replace each day with the average of cases around those days. All in all, we tried our best to correct the data only as strictly necessary, and as little as possible. The total cases we used are the ones reported on January 17, 2022. In the S1 File we include all the modified data, marked in yellow, to clarify the modifications we have made. The virus variants provide a dynamic scenario. We used the https://covariants.org/ (January 26, 2022) page as a reference of the variants evolution discussed for each country over time.

**Fig 2:**
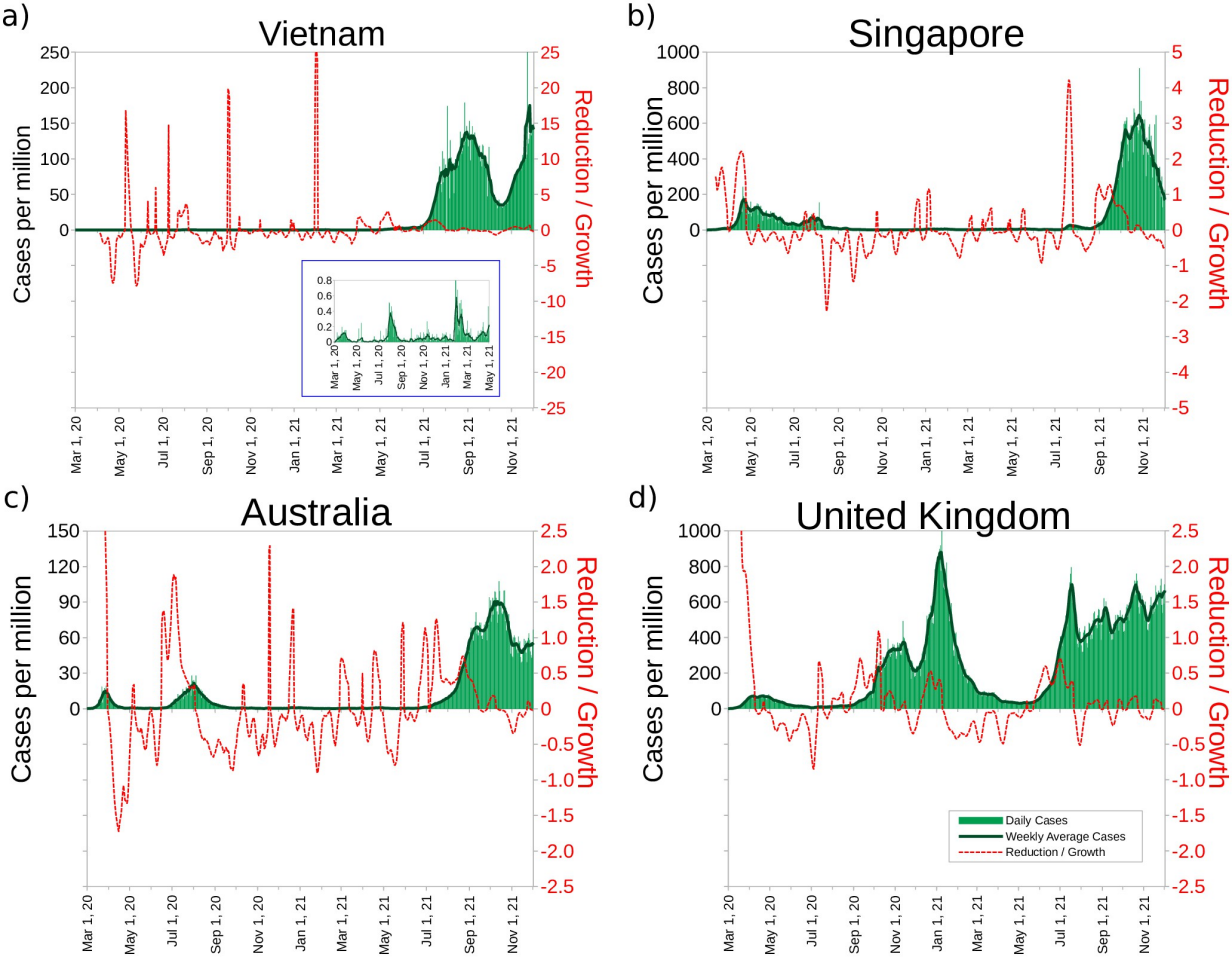
Illustration of the COVID19 evolution in four countries with different progressions. a) Vietnam b) Singapore c) Australia and d) United Kingdom.

**Fig 3:**
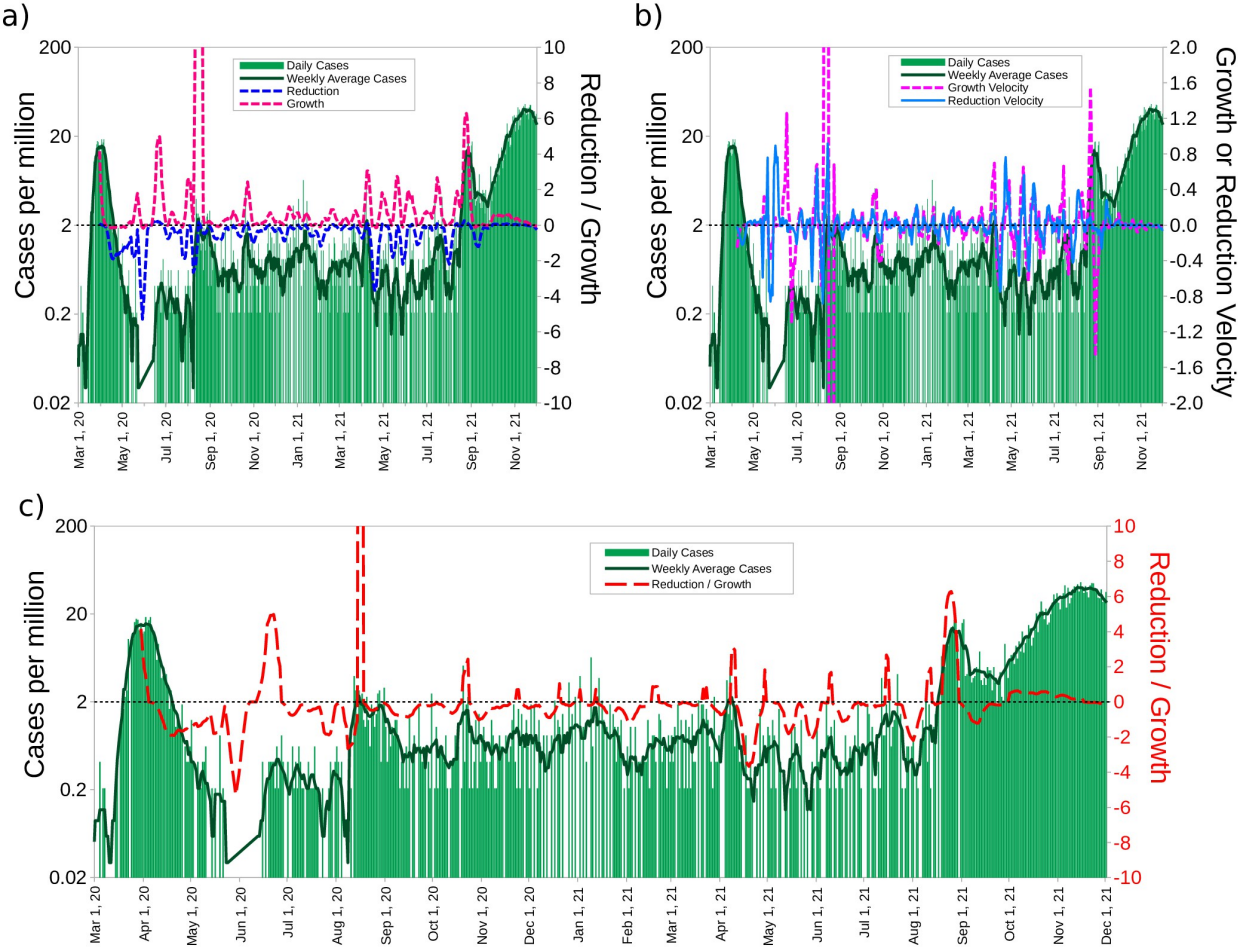
New Zealand. Time evolution of the variables. The date *t* = 0 is chosen throughout as the day the particular country exceeds 100 infections. a) Weekly averages of the number of cases, variation, reduction and growth as a function of time. Notice that the growth is many times larger than the reduction. b) Number of cases, their growth velocity and reduction velocity vs. time. c) Number of cases, their reduction/growth vs. time. New Zealand was able to react quickly to each wave before the arrival of the Delta variant. Starting in September 2021, New Zealand had a spike in cases that it was unable to control with the previous effectiveness.

**Fig 4:**
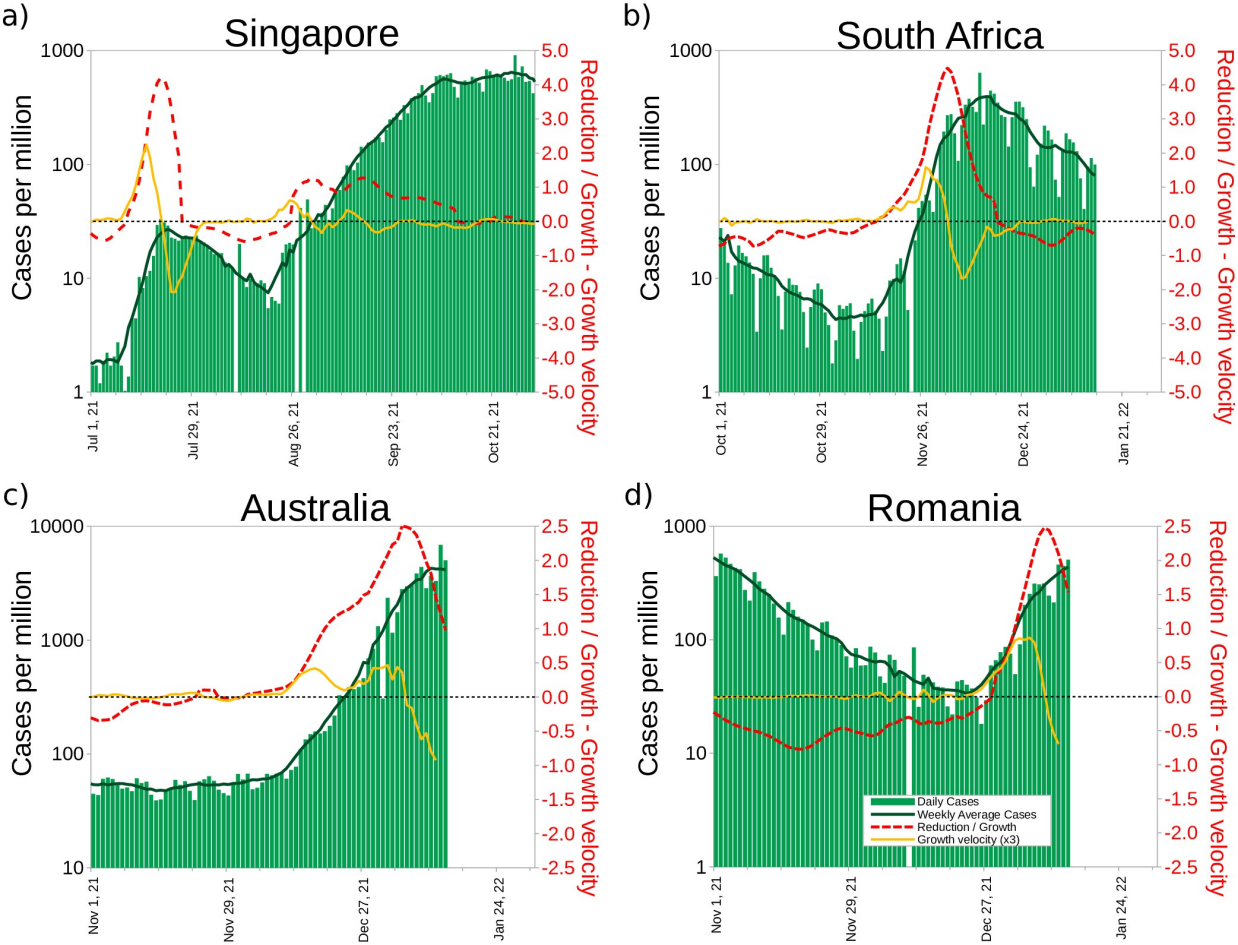
Illustration of the COVID-19 exponential growth moments. Velocity growth was multiplied by 3 to facilitate visualization on the same reduction/growth scale. a) Singapore b) South Africa c) Australia and d) Romania.

**Fig 5:**
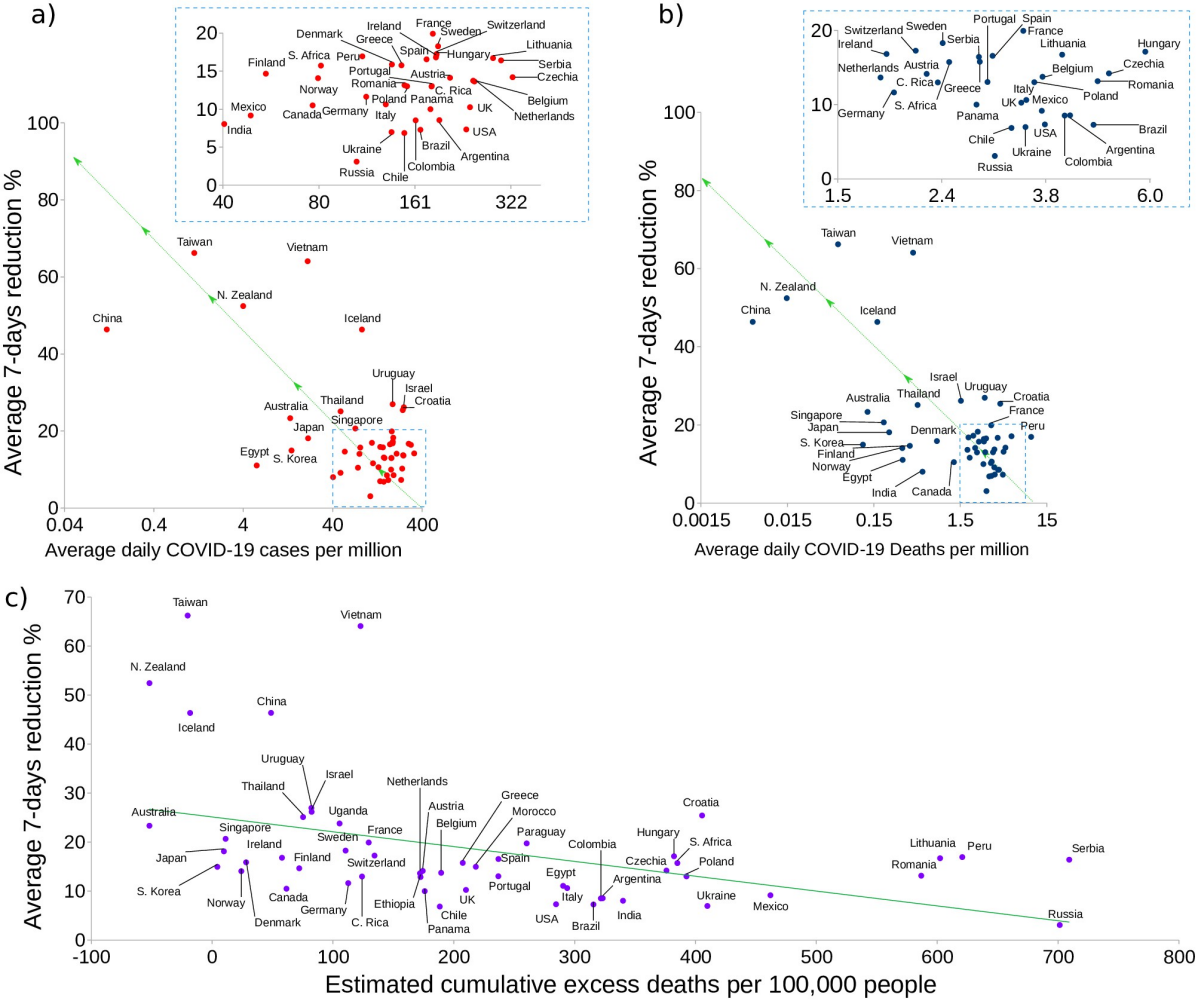
Data of 50 countries in scatter plots. a) Weekly average percentage of case reduction vs. average daily COVID-19 cases per million. Countries that have done well are consistently found upper left. b) Weekly average of the number of days COVID is reduced vs. average daily COVID-19 deaths per million; again we observe a similar country distribution as in a). c) Number of days where the reduction was large than 10% as vs. average daily COVID-19 deaths per million. There is a clear difference between the countries that have successfully controlled infections (upper left) and the ones that have not done so well (lower right). For each country, the daily average was calculated after they exceeded 100 accumulated cases.

**Fig 6:**
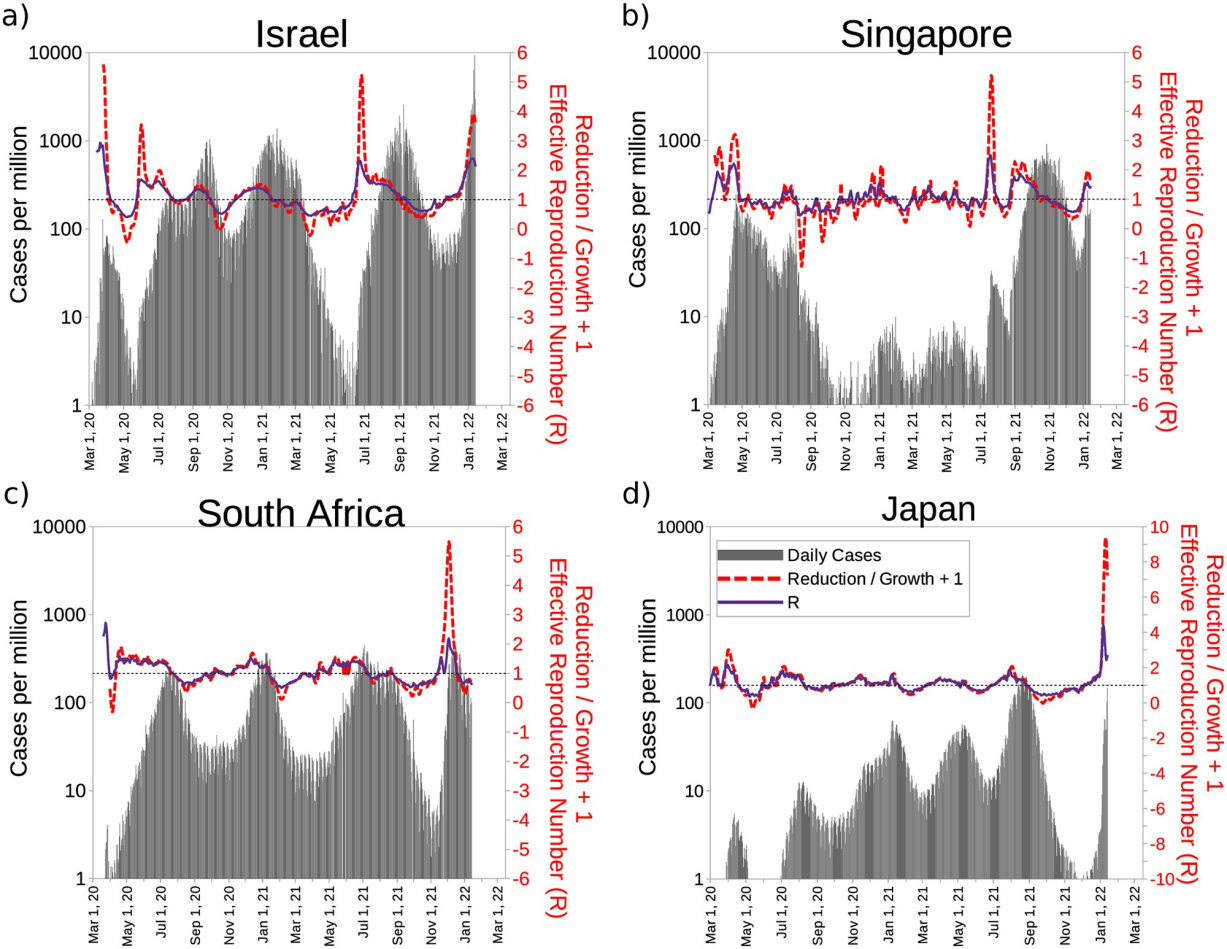
Comparison of the effective reproduction number (*R*) with the reduction/growth for four countries. a) Israel, b) Singapore, c) South Africa, and d) Japan. The dashed line the value *R* = 1, which indicates that contagion is expanding. When Reduction/growth is zero (*R*_*R*/*G*_ = 0) it coincides with *R* = 1; therefore, adding 1 to *R*_*R*/*G*_ both curves display similar behavior around *R* = 1.

The analysis presented here focuses mainly on what happened before Omicron. For this reason, the data used in Figs 2, 3 and 5 include information up to December 1, 2021. This point is critical to keep in mind for the comparison between countries that is presented in 5. In Figs 4 and 6, as in the figures of the S1 File, data are included up to the first days of January 2022.

## Results and discussion

Several different indicators have to be incorporated in the analysis to enhance the probability of effectively controlling COVID-19, but essentially what is required is to reduce the number of cases as fast as possible. When a new contagion focus is found, TTI or equivalent measures may be applied, but if they do not yield prompt results (*i.e.*, a significant reduction within 1–2 weeks), it means that the strategy will fail to control the contagion. Therefore, it is convenient to analyze the time variation of the variables above, *i.e.*, the time variation of the reduction.

To demonstrate the usefulness of these variables we examine four countries with different COVID-19 evolution as prototypes: Vietnam, Singapore, Australia, and the UK. To simplify the analysis and visualization, we plot in Fig 2 both growth and reduction as a single plot; that is, we plot *R*_*d*_(*t*) if *R*_*d*_(*t*)<0, and *G*(*t*) otherwise. Hereinafter, we will denote *R*_*R*/*G*_ to the combination of the reduction/growth as just defined.

At the time of this writing the Omicron variant is dominant around the world, and with the Omicron variants constantly appearing, we believe that it will take a few more months to draw appropriate conclusions about Omicron. Given that the case growth has been explosive, the recent data may distort our current analysis. Therefore, we analyze the data until December 1, 2021, when Omicron outbreaks began in earnest. Nevertheless, in the S1 File, we include the data for more than 50 countries until January 16.

We start with Vietnam, a successful crisis management example before May 2021, with a solid NPI that kept the total number of cases at 30 per million and the total number of deaths at 0.4 per million (35 deaths). A close examination of Fig 2a reveals that since March 2020, they have been able to maintain a strong reduction by reacting promptly to the infections rise. In fact, they managed to keep the reduction/growth *R*_*R*/*G*_, in the negative territory except for short growth periods, followed by a rapid reaction, with peaks below 1 case per million a day. From May 1, 2021 up to December 1, 2021 they faced their worst outbreak by far, reaching more than 150 cases per million inhabitants per day, and reporting an average over 350 deaths per day, around 10 times larger than their May 2021 cumulative figures. Also, *R*_*R*/*G*_ became positive at the beginning of May. It seems that everything that had worked previously with the Delta variant, did not work afterwards.

The evolution of Singapore is shown in Fig 2b, with an excellent performance until the end of June 2021. Furthermore, at the end of August 2021, they reached more than 75% population with a complete vaccination schedule. Although the Delta variant had become predominant in the country in May 2021, they managed to maintain control of the pandemic. At the beginning of July of that year, they had 36 cumulative confirmed deaths due to COVID-19. In July, strong case growth was observed for 15 consecutive days. This should explain the increase to 55 deaths accumulated at the beginning of September. Everything worsens from August 26, when 46 consecutive days of growth began. As a consequence, on December 1, 2021, they reported 726 accumulated deaths throughout the pandemic and increasing. In summary, more than 95% of Singaporean deaths have been reported during the second half of 2021. In S8 Fig in S1 File, we show the evolution of Singapore until mid-January 2022. The focus of our work is not on the effectiveness of vaccines, which have been tested in studies designed for that. What is clear from the Singaporean case is that vaccines alone are not sufficient to prevent large outbreaks, a factor that if ignored can have severe consequences. Therefore, NPI measures should be implemented in parallel with vaccination.

We now turn our attention to Australia, illustrated in Fig 2c, which was hit by COVID-19 in early March 2020. The reaction was swift, but the weekly average number of cases reached ∼15 per million by the end of March and was contained by the end of April. However, by mid-June of 2020 *R*_*R*/*G*_ became positive for 48 days, and the reaction was not speedy enough to prevent a second wave, which peaked at 21 cases per million in mid-August and extended to early October, but was under control until June 2021. By the end of June 2021 Delta became predominant in Australia, with about 10% of the population fully vaccinated. They suffered the worst outbreak in a year, and during many weeks the reduction was not driven into negative terrain. Actually, we count 86 days in a row without reduction since June 20, 2021. Until June 2021 Australia reported a cumulative of 910 confirmed COVID-19 deaths, that is, before the Delta variant. As of December 1, they had already exceeded 2,000 accumulated deaths. On this same date Australia had already reached more than 70% full vaccination. In S1, S2 Figs in S1 File we show Australia’s evolution until January 16, 2022, including the effects of the Omicron variant. In the last days of January 2022 there were over 3,150 confirmed deaths reported in Australia, and rising sharply. Even with partial data, the Omicron wave variant appears as the worst Australia has faced to date.

What happened in the United Kingdom is also worth a critical examination. The reduction/growth was in positive terrain *R*_*R*/*G*_ > 0 in early March 2021, and the number of cases per million *N*(*t*) grew to ∼100 per million by April 2021, without decreasing until the end of June. Unfortunately, in August *R*_*R*/*G*_ grew again, indicating that a second wave was approaching, which became quite serious by November.

Having reviewed what happened in Vietnam (a success story before the Delta variant), Singapore (a success until they lifted a lot of NPI measures), Australia (initial success followed by a second wave and long months of control up to the Delta variant), and the United Kingdom (where control of the situation was lost), we can now infer some general patterns about epidemic management.

The data examined above suggests that, if one pays close attention to the reduction/growth, two general conclusions can be drawn. The first inference is that keeping the reduction *R*_*d*_ constant is not sufficient; instead, one must aim toward the greatest reduction possible and pay attention when the variation of the reduction begins to increase. Theoretically, working on the largest reduction possible would quickly lead us to 0 cases, but in practice, we observed that in each outbreak *G* tends to be higher than any previous *R*_*d*_ rate, quickly returning to values of cases from previous weeks. Also, timely action is required as soon as the reduction *R*_*d*_ > 0. Below we discuss how to generalize these prescriptions by examining additional available data.

New Zealand has been singled out as a success story in handling COVID-19, perhaps even with the Delta variant as predominant, which proved to be very problematic for the countries we analyzed above. We now present a detailed study of what happened there, as illustrated in Fig 3, in the light of the methodology put forward above. There was a significant outbreak in March 2020, which prompted a vigorous reaction that reduced spectacularly, within a month, the number of cases, which then remained low for nearly two months. Thereafter, every time there was an outbreak, it was contained within a few days, as occurred with the August and October peaks. Each time a new outbreak, no matter how small, there was a swift response, mainly implementing TTI, with a strong effort to trace contacts, locate the infected and isolate them. A similar strategy was also followed in Iceland (See S4 Fig in S1 File). In Fig 3a, we observe that rapid action is advisable as soon as the reduction *R*_*d*_ > 0, and always keeping *R*_*d*_ < 0 for as long as possible. The dynamics are more evident when Fig 3b is inspected since the mid-August action and reaction are more apparent when the velocity is plotted vs. time. These conclusions are reinforced by Fig 3c, where we show that it is enough to follow the reduction and growth to analyze trends in a simple way as mentioned before (we plot *R*_*d*_ if *R*_*d*_ < 0, otherwise we plot *G*, *.i.e.*, *R*_*R*/*G*_).

Since the arrival of the Delta variant, the behavior of the New Zealand curve has changed. For the first time, *R*_*R*/*G*_ > 0 lasted for more than a month. This resulted in the highest case rise and a longer wave. In S7 Fig in S1 File, shows the evolution of New Zealand until January 16, 2022.

The case increase in New Zealand has had consequences. As of August 31, 2021, the country reported 26 COVID-19 confirmed deaths after about 17 months since the first case reported in the country. As of January 16, 2022, 3 and a half months later, it increased to 52 accumulated deaths.

The derivatives of the exponential function are themselves exponential functions, and we believe that in a scenario where cases, their variation and growth are all showing an upward trend, they are a clear indication that maximum attention is required. In Fig 4 we show four countries in which exponential growth appeared. The worst situation is sustained exponential growth for many days, and if this occurs, a country must work to quickly stop the spread. In Singapore, this clearly occurred during the July 2021 rise, where exponential growth was observed for about 2 weeks. With the appearance of the Omicron variant, the same thing happened in South Africa for about two weeks, during the second half of November. Similar behavior was observed in Australia and Romania due to this same reason.

In summary, we computed four variables and focused on their time evolution: case growth, reduction, variation and the velocity of each one of them. In particular, New Zealand’s growth velocity is, in general, significantly larger than the other two variables and allows for a rapid pinpointing of the instant case reduction occurs. Therefore, the reduction is central to our analysis and furthermore allows warning authorities and the population with a simple message: *if reduction stops, trouble lies ahead.*

We now turn to a more global outlook. The data plotted in Fig 5 for many countries is intended to show that our analysis is consistent with the worldwide evolution of COVID-19. We plot the average weekly reduction percentage (*R*_*d*_ as a percentage) vs. the logarithm of the average number of cases, in Fig 5a, and the logarithm of the average number of deaths in Fig 5b. The average is a daily average after the country reported more than 100 cumulative cases. The countries that have fared best are located on the top left while the worst performers are mostly found bottom right. Again it is quite apparent that a quick response and a prolonged time with a negative reduction (*R*_*d*_ < 0) yield better results. Additionally, we present the time evolution of cases and reduction/growth for 54 countries in the S1 File.

Reduction alone did not predict performance well enough in some countries, like Japan; additional indicators are required. We display some countries such as South Africa and India, which in the second quarter of 2021 have shown a sharp rise in cases and deaths. We also graph Israel’s case that, with the arrival of the Delta variant and despite a high percentage of vaccination, shows a strong case growth that should be a reminder that NPI measures and vaccination are needed together. It is quite likely that the intensive vaccination helped to reduce the number of deaths, but caution indicates that it is better to always act early.

It is important to point out that Fig 5 corresponds to a snapshot taken at a precise moment and varies in time; however, the average reduction evolves slowly enough to portray more than 40 countries in just one figure. These plots do not constitute a final verdict, but they allow to spot the cases that strongly deviate from the norm. They could be related to poor tracing [35, 36], different ways to keep statistics and/or sub-reporting.

Now we compare the evolution of the reduction/growth with the estimation of the effective reproduction number (*R*) calculated by Arroyo-Marioli *et al.* [37] and obtained from *ourworldindata.org* [33]. In the usual models, the epidemic is said to be expanding if *R* > 1. Above we showed that when reduction/growth *R*_*R*/*G*_ > 0 the situation is getting worse. If we add 1 to *R*_*R*/*G*_ we can compare it directly with *R*. In Fig 6 we compare the evolution of *R*_*R*/*G*_ + 1 vs. *R* for 4 countries. We observe that both curves coincide quite well for these 4 countries for values of *R* around 1. Our analysis for COVID-19 are quite comparable with this usual number in epidemiology. We believe it is prudent to emphasize that if *R*_*R*/*G*_ is used for other epidemics, a good indicator to compare should be *R*, to be sure that what we observed for COVID-19 can be transferred to new epidemics.

In the Fig 6 we show the evolution of 4 countries until the first days of January 2022. The appearance of the Omicron variant at the end of 2021 and the beginning of 2022 is quite apparent. *R*_*R*/*G*_ is calculated with respect to the situation 7 days before, and average smoothed with data of the previous 14 days. So it is a value related to recent history. This way a strong rise is observed in Israel in the mid-2021 with the arrival of the Delta variant, that even greater than what was observed later with the arrival of Omicron. In the latter case, the wave began when there was a much larger number of cases. On the other hand, *R*_*R*/*G*_ seems to us a motivating indicator when the situation improves. In this way, in Singapore a very pronounced reduction is observed by August 2020, which translates into a large negative value of *R*_*R*/*G*_ = −2.3. In contrast, *R* was just reduced to 0.54. To communicate to the population the value of reduction/growth also seems more motivating and simpler to explain.

Finally, it is important to emphasize that the indicators calculated here can be applied in the same way to the evolution of hospitalizations and deaths. The downside is that these are late indicators.

## Conclusions

Mitigating the impact of epidemic and achieving zero contagion is a primordial public health goal. Here we focus on the COVID-19 pandemic, but our analysis can be applied to other epidemic scenarios. Developing sound methodological tools to alert policymakers is essential to implement NPIs while vaccines are still under development, fabrication, and administration. Moreover, it has been shown that the effectiveness of vaccines is low in stopping contagion, being rather a factor in reducing severity and death. In this way, it seems almost impossible to achieve group immunity that allows preventing reactivation/mutation of the virus and new waves of contagion. Vaccines were at times considered a silver bullet for the COVID-19 pandemic. However, the lack of worldwide vaccination, the large anti-vaccine factions, and the emergence of new variants, which could lower vaccine effectiveness, have all led to more cautious expectations [38]. Moreover, amid the growing concern about new COVID variants [39–43], with renewed strong flu seasons, a short and mid-term estimate of evolution as the one presented here can help to guide public policy, independently of economic and cultural contexts.

We have presented performance indicators applicable to any NPI: reduction, growth, and variation (or velocities). These indicators complement other existing ones, such the effective reproduction number (*R*), and complex epidemic models [44, 45]. In fact, the application of these indicators to a large number of countries does allow early warning about a likely epidemic growth. We have identified conditions of maximum alert regarding epidemic evolution, when the cases, their variation and the growth velocity simultaneously increase.

Just above this Conclusion section, we compare the combined reduction/growth indicator *R*_*R*/*G*_ with the estimate of the effective reproduction number (*R*). We do not intend to replace the useful index *R* in epidemiology, but to point out advantages of *R*_*R*/*G*_ as far as explaining the health situation to the population, and to policy makers. Near *R* = 1 both indicators agree quite well. However, when contagion increases abruptly *R*_*R*/*G*_ grows strongly and *R* does not change significantly. On the other hand, when the situation improves significantly, while *R* varies between 0 and 1 (in a very favorable scenario it reaches ≈0.5), *R*_*R*/*G*_ drops strongly into negative terrain.

Obviously, experts do understand all cases correctly, but the interpretation of *R* seems more difficult to convey to the general population, since it involves knowledge of the behavior of exponential functions. Consequently, the concepts of reduction and growth are much easier to communicate to the wide public, and could help to explain why precaution or restrictive policies are working, or are necessary. Formally, both *R* and *R*_*R*/*G*_ are easily estimated.

To reach control of the epidemic, in addition to the NPI implemented and of the progress in the vaccination process, significant case reduction for extended periods is primordial. The indicators in this paper display country-wide behaviors, but can also be applied at a more local level, and might contribute to a better management of the epidemic when only a subpopulation, for example based on age or income, is included in the analysis. The indicators we examined are i) the seven day rolling reduction *R*_*d*_(*t*); ii) the growth *G*(*t*); iii) the growth velocity *V*(*t*) which describes the velocity of the variation of the number of contagions; and, iv) the velocity *v*_*G*_(*t*) with which the growth *G*(*t*) varies in time. Moreover, the loss of vaccine immunity with time together with emerging variants of the virus implies that additional tools to reduce the impact of a forthcoming pandemia are of crucial importance. Within the complex context of likely future events the indicators proposed here could offer some easy to implement guidelines to reduce epidemic impact, and they are formulated in a simple and direct fashion.

## Supporting information

S1 File (PDF)
