# Supplementary material for: Model based on COVID-19 evidence to predict and improve pandemic control
Source: PLoS One. 2023 Jun 15;18(6):e0286747. doi: 10.1371/journal.pone.0286747 (PMC10270358; doi:10.1371/journal.pone.0286747)
Supplement: S1 File — (PDF) [file pone.0286747.s001.pdf]

Supporting Information

Model based on COVID-19 evidence to predict and improve pandemic control

Rafael I. González[1,3], P. S. Moya[2], E. M. Bringa[4,1], G. Bacigalupe[6,7], M. Ramírez-Santana[8], and M. Kiwi*[3,2]

[1]Centro de Nanotecnología Aplicada, Universidad Mayor, Santiago, Chile.
[2]Departamento de Física, Facultad de Ciencias, Universidad de Chile, Santiago, Chile.
[3]Centro para el Desarrollo de la Nanociencia y la Nanotecnología, CEDENNA, Santiago, Chile.
[4]CONICET, Mendoza, Argentina.
[5]Universidad de Mendoza, Mendoza, Argentina.
[6]School of Education and Human Development, University of Massachusetts Boston, USA.
[7]CreaSur, Universidad de Concepción, Chile.
[8]Departamento de Salud Pública, Facultad de Medicina, Universidad Católica del Norte, Coquimbo, Chile.

email: m.kiwi.t@gmail.com

Contents

S1 Data plots for each country studied **S3**

S2 Modified data for some countries **S13**

S1 Data plots for each country studied

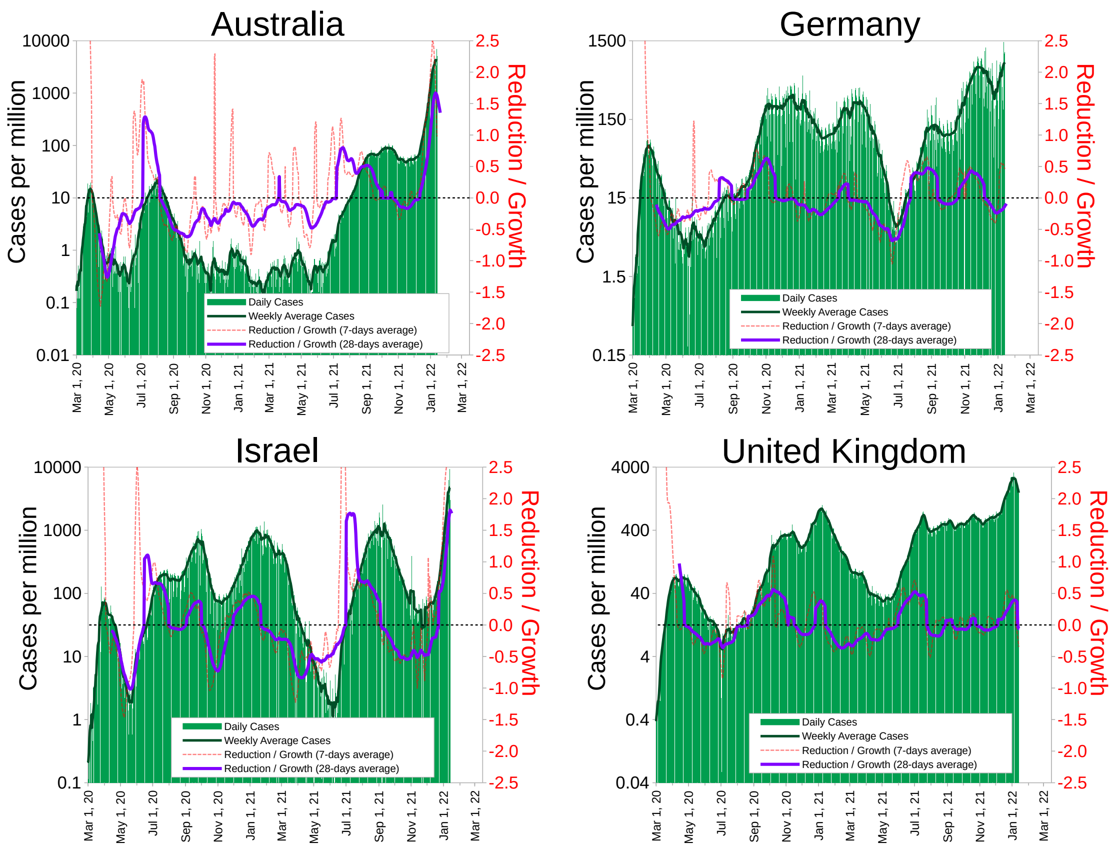

Fig S1. COVID-19 evolution in four countries with different progressions using 4 week moving averages Countries face problems when the reduction turns positive during many days. Here we show the reduction/growth average during a 4 week period. Many countries facing new outbreaks in the presence of the Delta variant. Australia, Germany, Israel and United Kingdom.

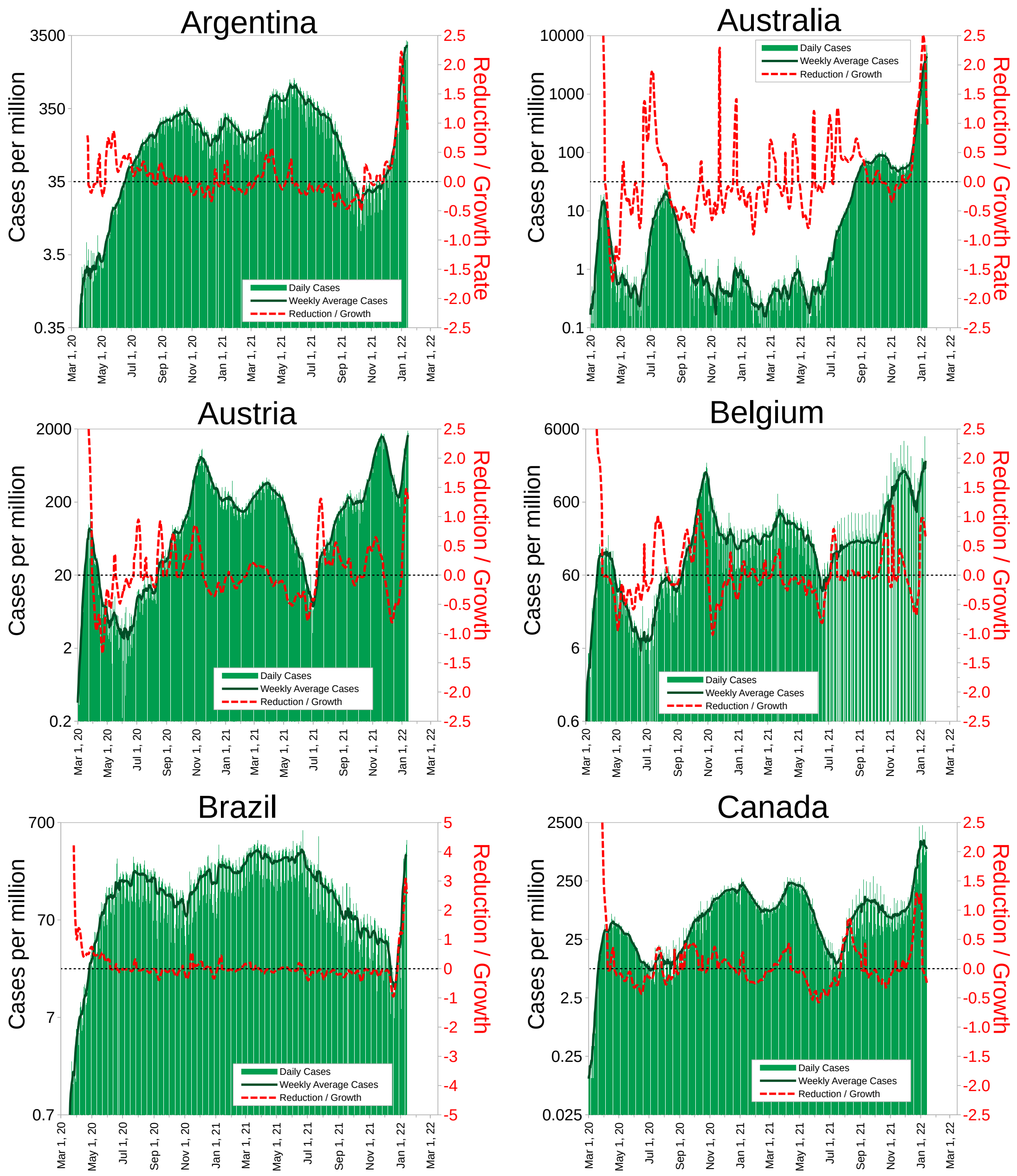

Fig S2. COVID-19 evolution: Argentina, Australia, Austria, Belgium, Brazil, Canada.

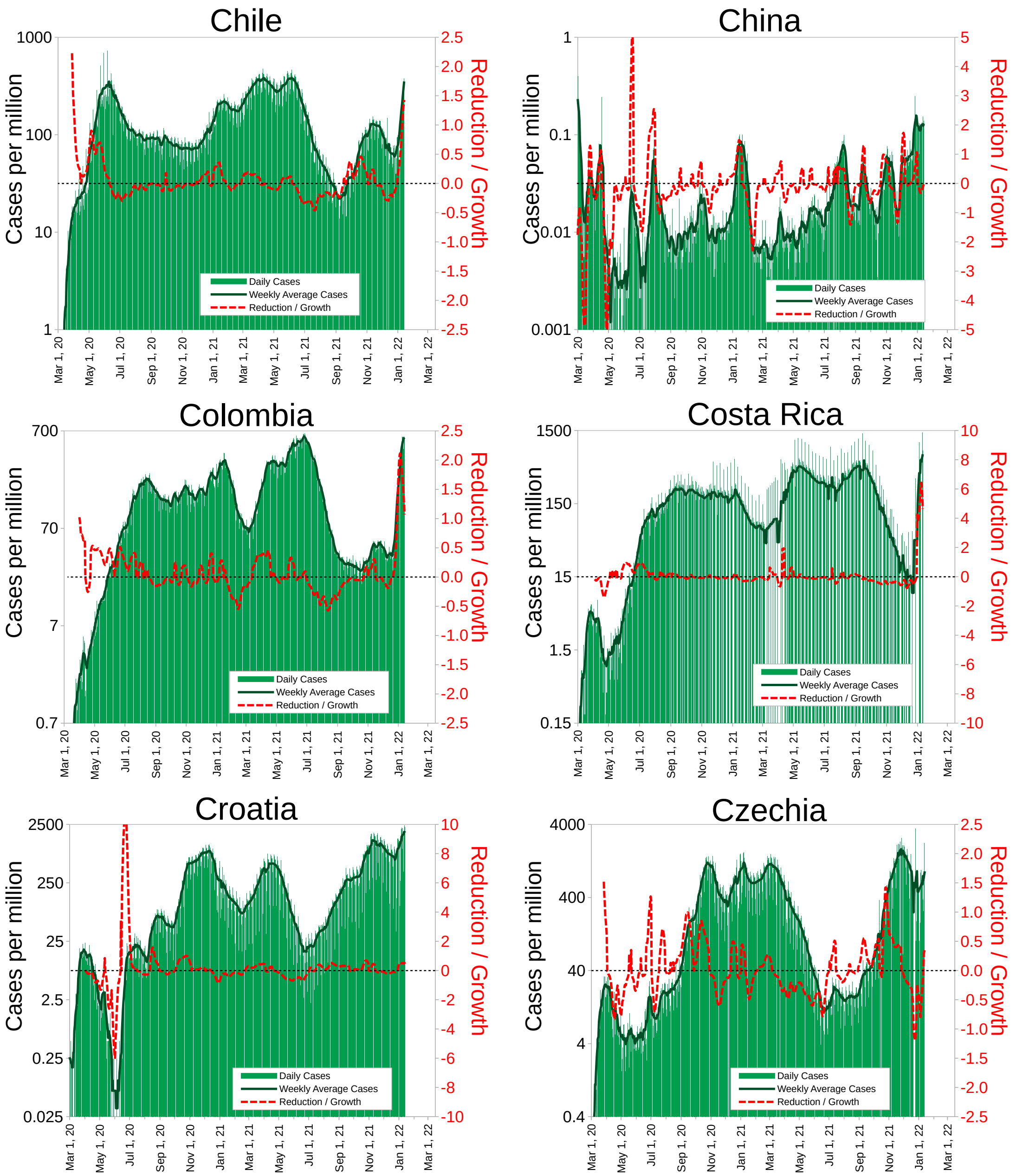

Fig S3. COVID-19 evolution: Chile, China, Colombia, Costa Rica, Croatia, Czechia.

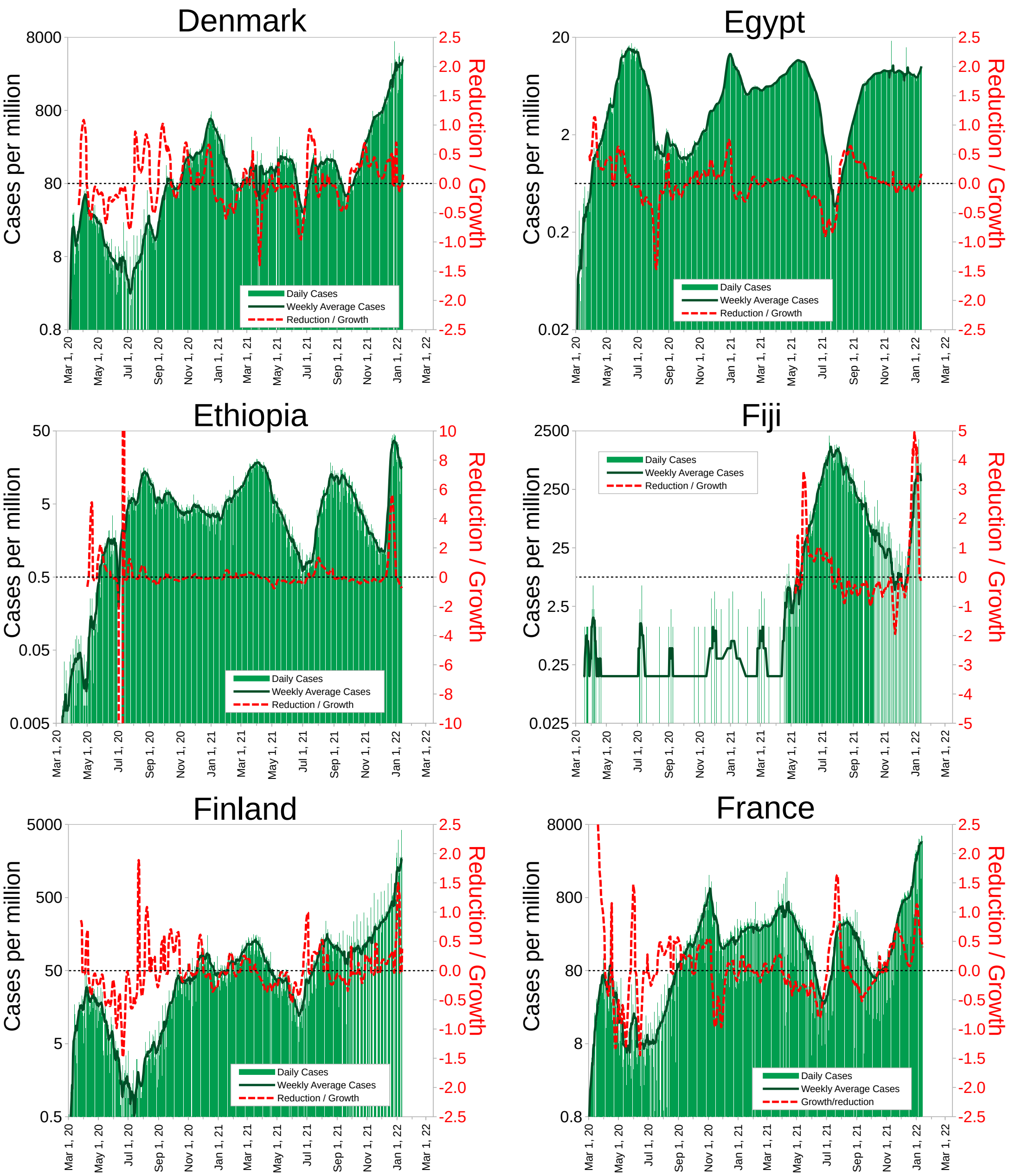

Fig S4. COVID-19 evolution: Denmark, Egypt, Ethiopia, Fiji, Finland, France.

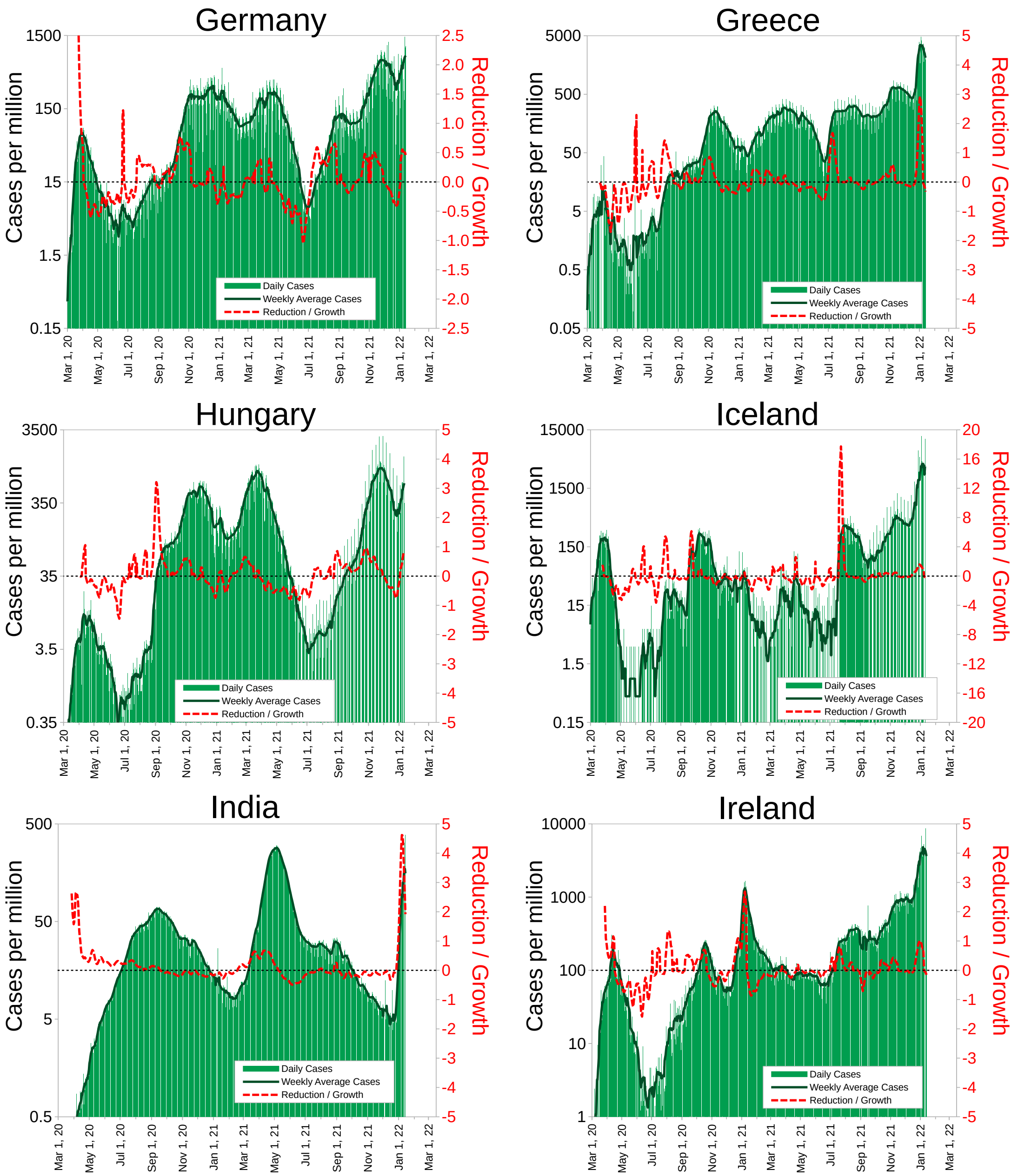

Fig S5. COVID-19 evolution: Germany, Greece, Hungary, Iceland, India, Ireland.

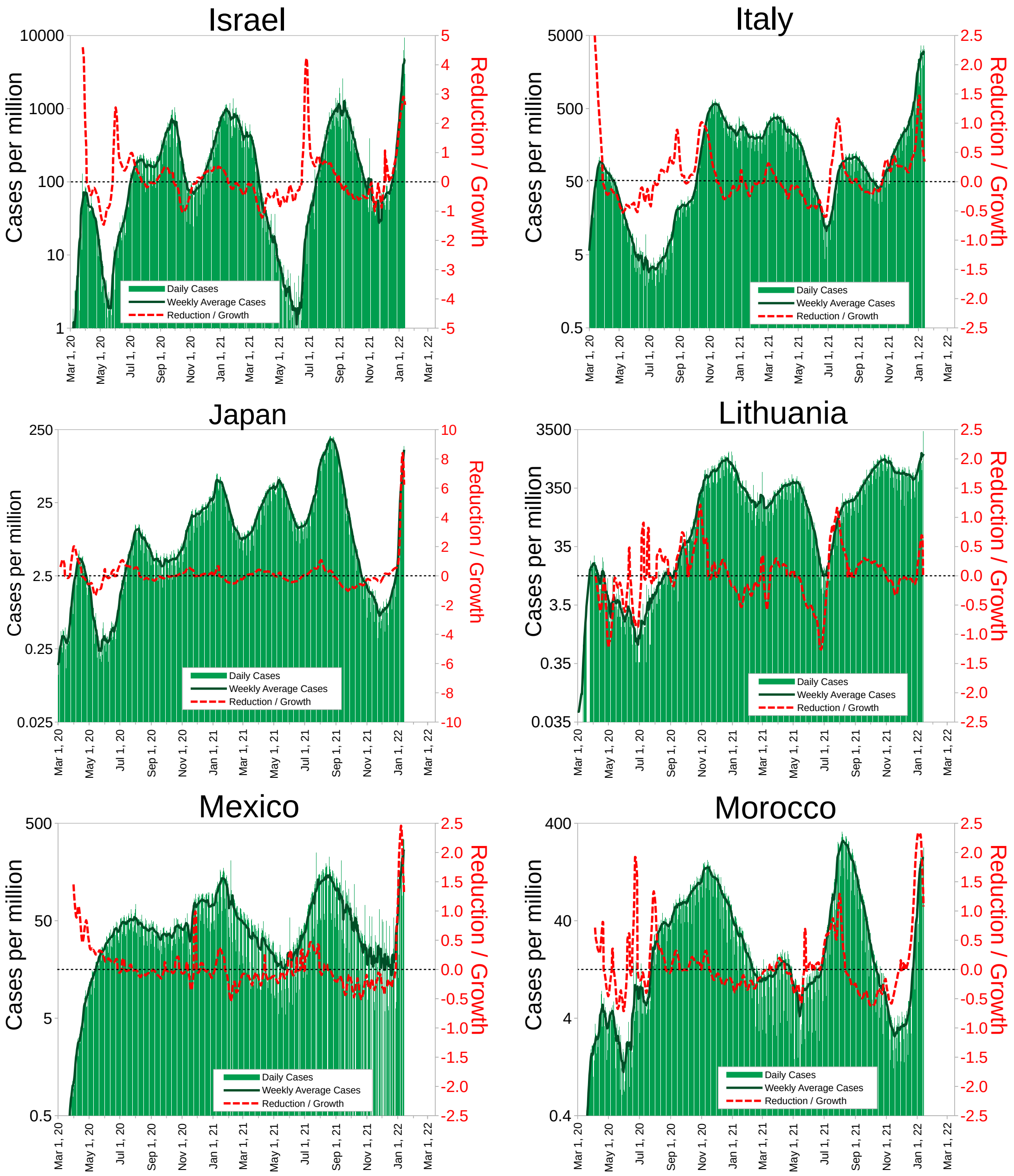

Fig S6. COVID-19 evolution: Israel, Italy, Japan, Lithuania, Mexico, Morocco.

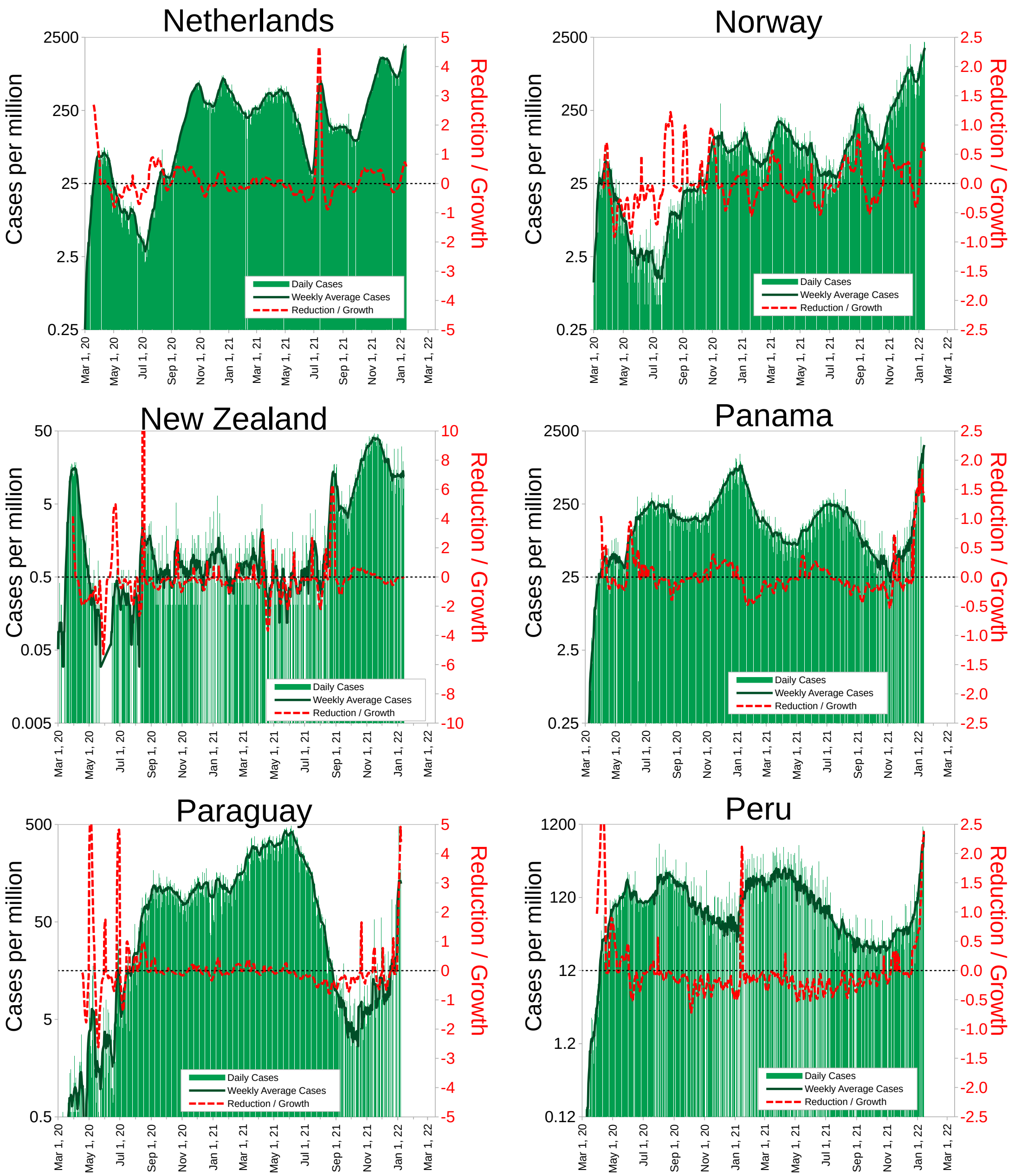

Fig S7. COVID-19 evolution: Netherlands, Norway, New Zealand, Panama, Paraguay, Peru.

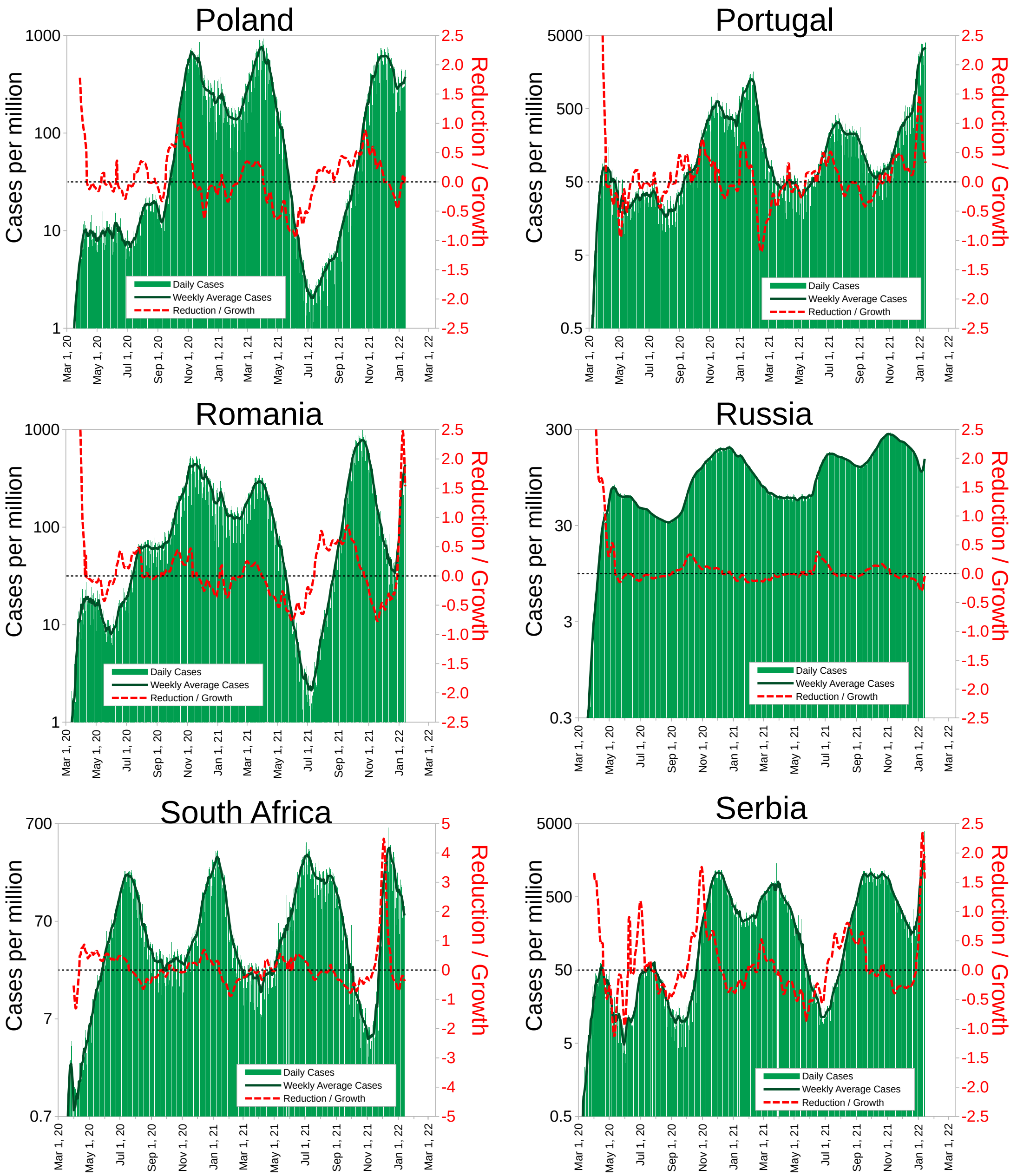

Fig S8. COVID-19 evolution: Poland, Portugal, Romania, Russia, South Africa, Serbia.

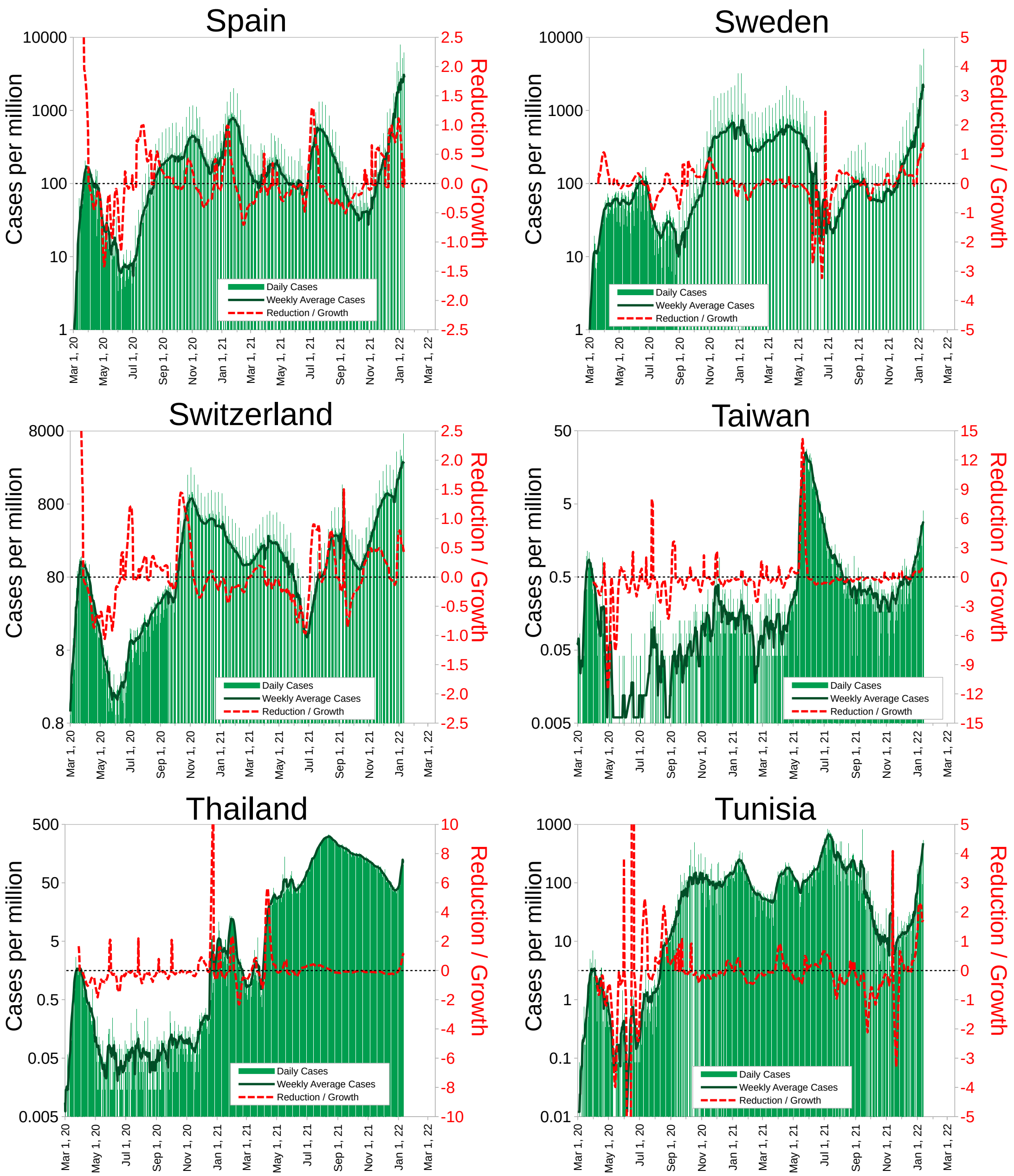

Fig S9. COVID-19 evolution: Spain, Sweden, Switzerland, Taiwan, Thailand, Tunisia.

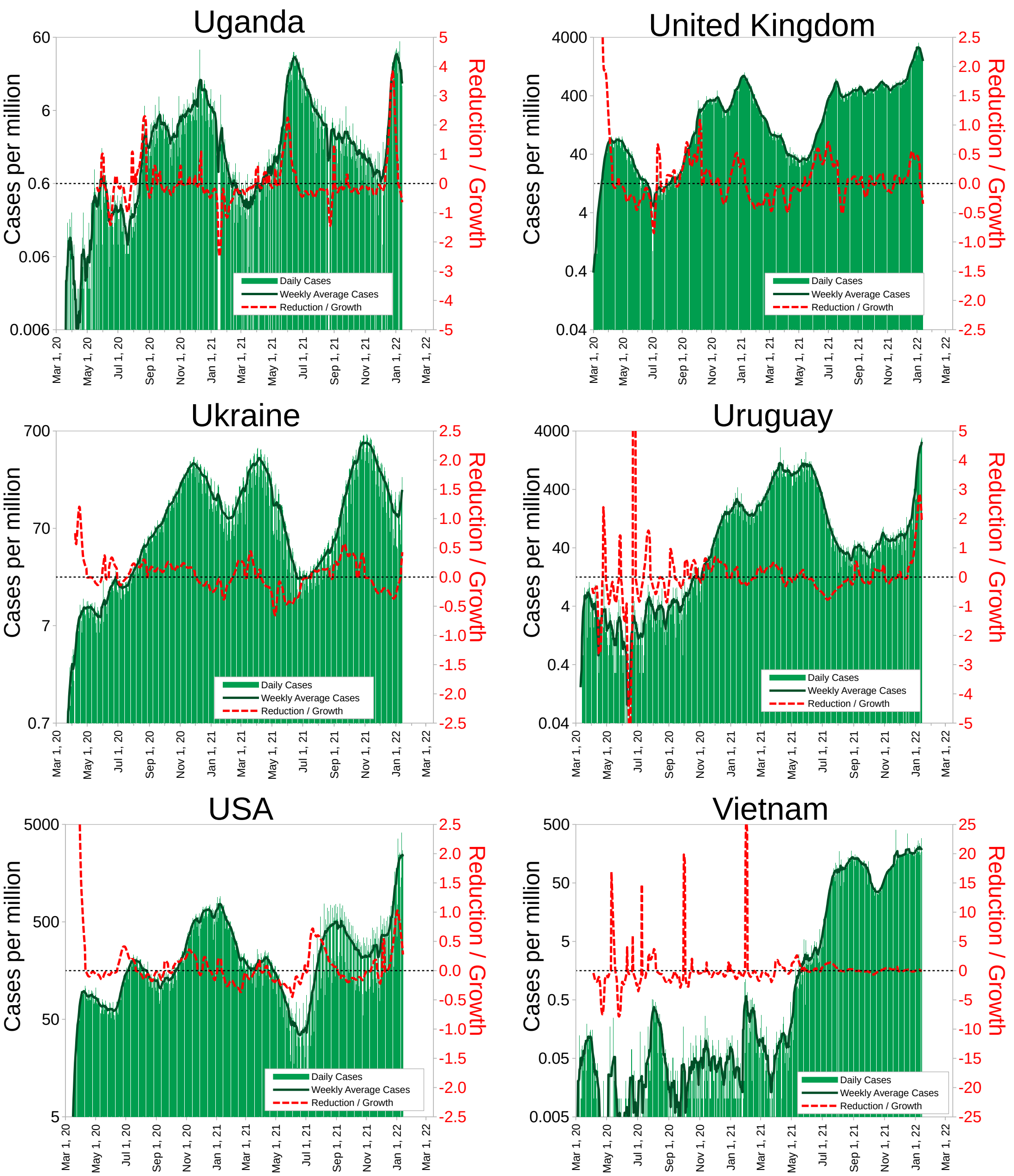

Fig S10. COVID-19 evolution: Uganda, United Kingdom, Ukraine, Uruguay, USA, Vietnam.

S2 Modified data for some countries

To calculate the "Average 7-day reduction %" for Figure 4 of the manuscript we modified a few data points that we understand to be anomalous, for 6 countries and in 2 cases. We replace all the negative values and the daily data that is 5 or more times larger than the previous 7 days average of (AP7D). In both cases we replace the original value by the AP7D. The modified data is detailed in Table S1, including the reasons we have to replace them. As can be verified by inspection of the values that are corrected, and the reasons to do so, their inclusion would lead to errors.

Table S1. Modified data values. The "Average of the Previous 7 Days" is abbreviated as AP7D.

Country	Date	Original value	Reason for modification
China	02/13/20	15133	6.1 times the AP7D
	06/03/20	-1	Negative value
France	04/04/20	-17074	Negative value
	04/07/20	-3491	Negative value
	04/12/20	50746	19,6 times the AP7D
	04/23/20	-1710	Negative value
	04/23/20	-1455	Negative value
	05/24/20	-439	Negative value
	06/02/20	-647	Negative value
	06/03/20	-3226	Negative value
	06/28/20	-406	Negative value
	11/04/20	-46076	Negative value
Mexico	10/05/20	28115	6.3 times the AP7D
Spain	04/24/20	-10034	Negative value
	05/25/20	-372	Negative value
	03/02/21	-74374	Negative value
Uganda	05/21/20	-104	Negative value
United Kingdom	04/09/21	-4787	Negative value
	05/18/21	-2362	Negative value

	China modified	China original	France modified	France original	Uganda modified	Uganda original	Mexico modified	Mexico original	Spain modified	Spain original	UK modified	UK original
01/23/20	92	92										
01/24/20	277	277	2	2								
01/25/20	483	483	1	1								
01/26/20	663	663	0	0								
01/27/20	801	801	0	0								
01/28/20	2,631	2,631	1	1								
01/29/20	576	576	1	1								
01/30/20	2,054	2,054	0	0								
01/31/20	1,659	1,659	0	0							2	2
02/01/20	2,088	2,088	1	1					1	1	0	0
02/02/20	4,736	4,736	0	0					0	0	0	0
02/03/20	3,086	3,086	0	0					0	0	6	6
02/04/20	3,987	3,987	0	0					0	0	0	0
02/05/20	3,729	3,729	0	0					0	0	1	1
02/06/20	3,144	3,144	0	0					0	0	0	0
02/07/20	3,522	3,522	0	0					0	0	0	0
02/08/20	2,703	2,703	5	5					0	0	4	4
02/09/20	3,012	3,012	0	0					1	1	1	1
02/10/20	2,516	2,516	0	0					0	0	0	0
02/11/20	2,021	2,021	0	0					0	0	1	1
02/12/20	372	372	0	0					0	0	1	1
02/13/20	2,470	15,133	0	0					0	0	1	1
02/14/20	6,460	6,460	0	0					0	0	1	1
02/15/20	2,055	2,055	1	1					0	0	0	0
02/16/20	2,099	2,099	0	0					0	0	0	0
02/17/20	1,918	1,918	0	0					0	0	1	1
02/18/20	1,775	1,775	0	0					0	0	0	0
02/19/20	407	407	0	0					0	0	1	1
02/20/20	453	453	0	0					0	0	2	2
02/21/20	473	473	0	0					0	0	1	1
02/22/20	1,450	1,450	0	0					0	0	0	0
02/23/20	16	16	0	0					0	0	5	5
02/24/20	214	214	0	0					0	0	2	2
02/25/20	508	508	2	2					4	4	4	4
02/26/20	405	405	4	4					7	7	3	3
02/27/20	433	433	20	20					2	2	7	7
02/28/20	326	326	19	19			1	1	17	17	12	12
02/29/20	427	427	43	43			3	3	13	13	5	5
03/01/20	575	575	30	30			1	1	39	39	33	33
03/02/20	200	200	61	61			0	0	36	36	40	40
03/03/20	125	125	21	21			0	0	45	45	55	55
03/04/20	120	120	76	76			0	0	57	57	56	56
03/05/20	151	151	138	138			0	0	37	37	49	49
03/06/20	151	151	190	190			1	1	141	141	79	79
03/07/20	79	79	332	332			0	0	100	100	55	55
03/08/20	47	47	177	177			1	1	173	173	54	54
03/09/20	36	36	286	286			0	0	400	400	147	147
03/10/20	22	22	372	372			0	0	622	622	259	259
03/11/20	28	28	510	510			1	1	582	582	412	412
03/12/20	8	8	0	0			4	4	0	0	489	489
03/13/20	8	8	1,388	1,385			14	14	2,955	2,955	479	479

03/14/20	26	26	815	815			15	15	1,159	1,159	364	364
03/15/20	21	21	36	36			12	12	1,407	1,407	443	443
03/16/20	19	19	2,151	2,151			29	29	2,144	2,144	615	615
03/17/20	17	17	1,032	1,032			11	11	1,806	1,806	768	768
03/18/20	22	22	1,409	1,409			25	25	2,162	2,162	999	999
03/19/20	25	25	1,846	1,841			46	46	4,053	4,053	1,060	1,060
03/20/20	46	46	1,788	1,783			39	39	2,447	2,447	1,257	1,257
03/21/20	37	37	1,705	1,699	1	1	48	48	4,964	4,964	1,215	1,215
03/22/20	80	80	2,295	2,295	0	0	65	65	3,394	3,394	1,376	1,376
03/23/20	24	24	3,365	3,355	8	8	51	51	6,368	6,368	2,339	2,339
03/24/20	62	62	2,249	2,243	0	0	38	38	4,749	4,749	2,371	2,371
03/25/20	41	41	2,962	2,955	5	5	70	70	9,630	9,630	2,703	2,703
03/26/20	77	77	3,918	3,916	0	0	110	110	8,271	8,271	3,104	3,104
03/27/20	47	47	3,795	3,792	9	9	132	132	7,933	7,933	3,223	3,223
03/28/20	56	56	4,645	4,648	7	7	131	131	7,516	7,516	2,831	2,831
03/29/20	43	43	2,603	2,603	3	3	145	145	6,875	6,875	2,868	2,868
03/30/20	34	34	4,354	4,343	0	0	101	101	7,846	7,846	4,306	4,306
03/31/20	46	46	7,629	7,635	11	11	121	121	7,967	7,967	4,515	4,515
04/01/20	31	31	4,844	4,843	0	0	163	163	8,195	8,195	4,945	4,945
04/02/20	34	34	2,106	2,100	1	1	132	132	7,947	7,947	4,886	4,886
04/03/20	34	34	5,224	5,223	3	3	178	178	7,134	7,134	4,954	4,954
04/04/20	15	15	4,486	-17,076	0	0	202	202	6,969	6,969	4,050	4,050
04/05/20	30	30	855		4	4	253	253	5,478	5,478	3,641	3,641
04/06/20	39	39	2,654	2,658	0	0	296	296	5,029	5,029	5,289	5,289
04/07/20	32	32	3,971	-3,494	0	0	346	346	5,267	5,267	5,462	5,462
04/08/20	65	65	3,855	3,851	1	1	396	396	6,278	6,278	5,145	5,145
04/09/20	61	61	3,784	3,784	0	0	260	260	5,002	5,002	4,862	4,862
04/10/20	42	42	1,565	1,566	0	0	403	403	5,051	5,051	4,352	4,352
04/11/20	62	62	1,445	1,445	0	0	375	375	4,754	4,754	3,586	3,586
04/12/20	116	116	2,590	50,745	1	1	442	442	3,804	3,804	3,519	3,519
04/13/20	74	74	3,141	3,139	0	0	353	353	3,268	3,268	4,188	4,188
04/14/20	90	90	17,448	17,448	1	1	385	385	2,442	2,442	4,329	4,329
04/15/20	45	45	3,208	3,207	0	0	448	448	5,103	5,103	5,072	5,072
04/16/20	47	47	12,578	12,578	0	0	450	450	7,304	7,304	5,308	5,308
04/17/20	353	353	1,891	1,891	1	1	578	578	5,891	5,891	4,978	4,978
04/18/20	24	24	3,806	3,804	0	-1	622	622	887	887	4,730	4,730
04/19/20	17	17	1,092	1,094	0	0	764	764	6,948	6,948	3,856	3,856
04/20/20	12	12	2,447	2,446	1	1	511	511	1,536	1,536	4,858	4,858
04/21/20	32	32	2,666	2,666	5	5	729	729	3,968	3,968	4,768	4,768
04/22/20	11	11	1,800	1,798	2	2	1,043	1,043	4,211	4,211	5,492	5,492
04/23/20	14	14	3,754	-1,709	11	11	1,089	1,089	4,635	4,635	5,166	5,166
04/24/20	15	15	2,811	2,811	1	1	1,239	1,239	4,011	-10,034	4,975	4,975
04/25/20	8	8	1,678	1,678	0	0	970	970	2,915	2,915	3,750	3,750
04/26/20	3	3	633	633	4	4	835	835	1,729	1,729	3,468	3,468
04/27/20	6	6	3,686	3,686	0	0	852	852	1,831	1,831	4,702	4,702
04/28/20	22	22	3,132	3,131	0	0	1,223	1,223	1,308	1,308	4,726	4,726
04/29/20	4	4	2,499	-1,455	2	2	1,047	1,047	2,144	2,144	5,442	5,442
04/30/20	12	12	1,218	1,218	2	2	1,425	1,425	518	518	4,956	4,956
05/01/20	1	1	526	526	2	2	1,515	1,515	1,781	1,781	4,728	4,728
05/02/20	0	0	792	792	3	3	1,349	1,349	1,366	1,366	3,226	3,226
05/03/20	5	5	361	361	1	1	1,383	1,383	884	884	2,975	2,975
05/04/20	1	1	562	562	8	8	1,434	1,434	545	545	3,378	3,378
05/05/20	2	2	1,117	1,115	1	1	1,120	1,120	1,318	1,318	3,685	3,685

05/06/20	2	2	4,136	4,136	2	2	1,609	1,609	996	996	3,831	3,831
05/07/20	1	1	741	741	1	1	1,982	1,982	1,122	1,122	3,767	3,767
05/08/20	1	1	531	531	0	0	1,906	1,906	1,410	1,410	3,049	3,049
05/09/20	14	14	529	528	15	15	1,938	1,938	721	721	2,150	2,150
05/10/20	17	17	249	249	5	5	1,562	1,562	772	772	2,321	2,321
05/11/20	1	1	314	315	0	0	1,305	1,305	3,086	3,086	3,584	3,584
05/12/20	7	7	753	753	8	8	1,997	1,997	594	594	3,390	3,390
05/13/20	3	3	521	521	10	10	1,862	1,862	661	661	3,299	3,299
05/14/20	4	4	642	642	21	21	2,409	2,409	849	849	2,610	2,610
05/15/20	8	8	513	513	43	43	2,437	2,437	643	643	2,517	2,517
05/16/20	6	6	415	415	24	24	2,112	2,112	515	515	2,070	2,070
05/17/20	7	7	140	140	0	0	2,075	2,075	0	0	1,826	1,826
05/18/20	9	9	444	444	21	21	2,414	2,414	908	908	2,575	2,575
05/19/20	0	0	501	501	12	12	2,713	2,713	431	431	3,041	3,041
05/20/20	0	0	482	481	4	4	2,248	2,248	518	518	2,709	2,709
05/21/20	0	0	267	267	18	-104	2,973	2,973	482	482	2,542	2,542
05/22/20	8	8	756	756	15	15	2,960	2,960	1,787	1,787	2,030	2,030
05/23/20	3	3	546	546	23	23	3,329	3,329	466	466	1,507	1,507
05/24/20	11	11	448	-439	0	0	2,764	2,764	482	482	1,348	1,348
05/25/20	7	7	325	325	24	24	2,485	2,485	725	-372	1,608	1,608
05/26/20	1	1	255	255	31	31	3,455	3,455	859	859	1,650	1,650
05/27/20	2	2	172	173	28	28	3,463	3,463	0	0	1,803	1,803
05/28/20	0	0	3,343	3,342	36	36	3,377	3,377	1,647	1,647	1,734	1,734
05/29/20	4	4	594	594	12	12	3,227	3,227	658	658	1,504	1,504
05/30/20	2	2	1,795	1,794	84	84	2,885	2,885	664	664	1,099	1,099
05/31/20	16	16	231	232	4	4	3,152	3,152	251	251	1,069	1,069
06/01/20	5	5	407	406	40	40	2,771	2,771	159	159	1,434	1,434
06/02/20	1	1	971	-647	32	32	3,891	3,891	294	294	1,467	1,467
06/03/20	4	-1	1,073	-3,226	18	18	3,912	3,912	394	394	1,343	1,343
06/04/20	5	5	4,360	4,360	15	15	4,442	4,442	334	334	1,234	1,234
06/05/20	3	3	581	581	35	35	4,346	4,346	318	318	1,105	1,105
06/06/20	6	6	572	572	36	36	3,593	3,593	332	332	793	793
06/07/20	4	4	341	341	23	23	3,484	3,484	240	240	717	717
06/08/20	3	3	274	274	30	30	2,999	2,999	167	167	1,087	1,087
06/09/20	3	3	553	552	11	11	4,199	4,199	249	249	1,154	1,154
06/10/20	11	11	625	625	8	8	4,883	4,883	314	314	1,182	1,182
06/11/20	7	7	364	364	14	14	4,790	4,790	427	427	1,001	1,001
06/12/20	11	11	802	801	7	7	5,222	5,222	502	502	1,053	1,053
06/13/20	57	57	497	497	8	8	3,494	3,494	396	396	887	887
06/14/20	49	49	390	390	2	2	4,147	4,147	323	323	807	807
06/15/20	40	40	136	136	9	9	3,427	3,427	181	181	1,029	1,029
06/16/20	44	44	663	663	19	19	4,599	4,599	219	219	1,077	1,077
06/17/20	28	28	488	489	8	8	4,930	4,930	355	355	999	999
06/18/20	32	32	586	586	9	9	5,662	5,662	585	585	1,020	1,020
06/19/20	0	0	787	787	14	14	5,030	5,030	307	307	980	980
06/20/20	55	55	619	619	8	8	4,717	4,717	363	363	666	666
06/21/20	16	16	343	343	7	7	5,343	5,343	334	334	622	622
06/22/20	22	22	142	142	4	4	4,577	4,577	232	232	892	892
06/23/20	13	13	698	698	23	23	6,288	6,288	248	248	882	882
06/24/20	18	18	268	268	8	8	5,437	5,437	334	334	775	775
06/25/20	13	13	255	255	16	16	6,104	6,104	400	400	697	697
06/26/20	21	21	1,263	1,263	12	12	5,441	5,441	419	419	638	638
06/27/20	17	17	448	448	15	15	4,410	4,410	564	564	633	633

06/28/20	12	12	488	-409	11	11	4,050	4,050	301	301	395	395
06/29/20	19	19	645	645	11	11	3,805	3,805	200	200	604	604
06/30/20	3	3	441	442	19	19	5,432	5,432	301	301	403	403
07/01/20	3	3	990	990	4	4	5,681	5,681	388	388	60	60
07/02/20	5	5	539	539	9	9	6,741	6,741	444	444	4	4
07/03/20	3	3	539	539	9	9	6,740	6,740	442	442	502	502
07/04/20	8	8	358	358	16	16	6,914	6,914	0	0	624	624
07/05/20	4	4	11	12	12	12	4,683	4,683	0	0	516	516
07/06/20	1	1	1,018	1,018	14	14	4,902	4,902	1,244	1,244	358	358
07/07/20	14	14	454	454	18	18	6,258	6,258	341	341	581	581
07/08/20	9	9	822	822	6	6	6,995	6,995	383	383	630	630
07/09/20	0	0	444	444	23	23	7,280	7,280	543	543	642	642
07/10/20	0	0	715	714	6	6	6,891	6,891	852	852	512	512
07/11/20	13	13	251	251	7	7	6,094	6,094	0	0	820	820
07/12/20	8	8	16	16	12	12	4,482	4,482	0	0	650	650
07/13/20	0	0	1,423	1,423	4	4	4,685	4,685	2,045	2,045	534	534
07/14/20	9	9	62	62	11	11	7,051	7,051	666	666	1,240	1,240
07/15/20	1	1	727	726	3	3	6,149	6,149	875	875	538	538
07/16/20	14	14	569	569	8	8	6,406	6,406	1,361	1,361	643	643
07/17/20	17	17	829	824	5	5	7,257	7,257	1,400	1,400	687	687
07/18/20	16	16	163	165	6	6	7,615	7,615	0	0	827	827
07/19/20	21	21	4	4	3	3	5,311	5,311	0	0	726	726
07/20/20	11	11	1,911	1,911	4	4	5,172	5,172	4,581	4,581	580	580
07/21/20	13	13	595	595	3	3	6,859	6,859	1,358	1,358	445	445
07/22/20	25	25	921	921	3	3	6,019	6,019	1,357	1,357	560	560
07/23/20	21	21	1,236	1,236	4	4	8,438	8,438	2,615	2,615	769	769
07/24/20	34	34	1,091	1,091	10	10	7,573	7,573	2,255	2,255	769	769
07/25/20	46	46	88	84	14	14	6,751	6,751	0	0	767	767
07/26/20	61	61	0	4	12	12	5,480	5,480	0	0	745	745
07/27/20	68	68	2,488	2,488	13	13	4,973	4,973	6,361	6,361	685	685
07/28/20	101	101	564	564	7	7	7,208	7,208	1,828	1,828	550	550
07/29/20	105	105	1,470	1,470	5	5	5,752	5,752	2,031	2,031	797	797
07/30/20	127	127	1,389	1,394	7	7	7,730	7,730	2,789	2,789	846	846
07/31/20	45	45	1,308	1,303	7	7	8,458	8,458	3,092	3,092	880	880
08/01/20	48	48	63	62	22	22	9,556	9,556	0	0	761	761
08/02/20	43	43	3	3	6	6	4,853	4,853	0	0	743	743
08/03/20	36	36	3,360	3,361	13	13	4,767	4,767	8,532	8,532	942	942
08/04/20	27	27	971	969	8	8	6,148	6,148	5,760	5,760	674	674
08/05/20	37	37	1,708	1,708	10	10	6,139	6,139	2,953	2,953	892	892
08/06/20	37	37	1,601	1,601	10	10	6,590	6,590	4,088	4,088	951	951
08/07/20	31	31	2,387	2,382	31	31	6,717	6,717	4,507	4,507	871	871
08/08/20	23	23	70	69	13	13	6,495	6,495	0	0	758	758
08/09/20	49	49	35	6	16	16	4,376	4,376	0	0	1,062	1,062
08/10/20	44	44	4,731	4,717	14	14	5,558	5,558	8,618	8,618	818	818
08/11/20	19	19	1,414	1,408	16	16	6,686	6,686	3,632	3,632	1,148	1,148
08/12/20	25	25	2,598	2,577	19	19	5,858	5,858	3,172	3,172	1,013	1,013
08/13/20	30	30	2,678	2,670	21	21	7,371	7,371	7,550	7,550	1,129	1,129
08/14/20	22	22	2,923	2,887	32	32	5,618	5,618	5,479	5,479	1,444	1,444
08/15/20	19	19	3,210	3,210	49	49	6,345	6,345	0	0	1,012	1,012
08/16/20	22	22	3,003	3,020	66	66	4,448	4,448	0	0	1,105	1,105
08/17/20	22	22	650	590	60	60	3,571	3,571	16,269	16,269	715	715
08/18/20	17	17	2,119	2,118	43	43	5,506	5,506	5,114	5,114	1,089	1,089
08/19/20	7	7	3,772	3,787	53	53	5,792	5,792	6,671	6,671	816	816

08/20/20	22	22	4,800	4,800	94	94	6,775	6,775	7,039	7,039	1,182	1,182
08/21/20	22	22	5,009	4,954	98	98	5,928	5,928	8,148	8,148	1,034	1,034
08/22/20	12	12	3,474	3,474	318	318	6,482	6,482	0	0	1,288	1,288
08/23/20	16	16	4,680	4,735	97	97	3,948	3,948	0	0	1,041	1,041
08/24/20	14	14	1,976	1,976	99	99	3,541	3,541	19,382	19,382	974	974
08/25/20	15	15	3,616	3,432	64	64	4,916	4,916	7,117	7,117	1,188	1,188
08/26/20	8	8	287	244	98	98	5,267	5,267	7,296	7,296	1,051	1,051
08/27/20	9	9	11,203	11,226	155	155	6,026	6,026	9,658	9,658	1,522	1,522
08/28/20	9	9	7,641	7,558	77	77	5,824	5,824	9,779	9,779	1,278	1,278
08/29/20	9	9	5,460	5,460	91	91	5,974	5,974	0	0	1,108	1,108
08/30/20	17	17	5,366	5,382	81	81	4,129	4,129	0	0	1,715	1,715
08/31/20	10	10	3,113	3,022	44	44	3,719	3,719	23,572	23,572	1,406	1,406
09/01/20	7	7	5,104	5,104	65	65	6,476	6,476	8,115	8,115	1,300	1,300
09/02/20	12	12	7,113	7,038	75	75	4,921	4,921	8,581	8,581	1,509	1,509
09/03/20	25	25	7,122	7,119	176	176	5,937	5,937	8,959	8,959	1,735	1,735
09/04/20	10	10	8,834	8,788	65	65	6,196	6,196	10,476	10,476	1,940	1,940
09/05/20	10	10	8,594	8,594	186	186	6,319	6,319	0	0	1,813	1,813
09/06/20	12	12	7,016	7,015	128	128	4,614	4,614	0	0	2,983	2,983
09/07/20	9	9	4,266	4,184	109	109	3,486	3,486	26,560	26,560	2,950	2,950
09/08/20	3	3	6,738	6,738	124	124	5,351	5,351	8,964	8,964	2,461	2,461
09/09/20	7	7	8,619	8,577	201	201	4,461	4,461	8,866	8,866	2,661	2,661
09/10/20	15	15	9,762	9,699	190	190	5,043	5,043	10,764	10,764	2,919	2,919
09/11/20	6	6	9,509	9,433	86	86	5,935	5,935	12,183	12,183	3,541	3,541
09/12/20	10	10	11,337	11,336	326	326	5,674	5,674	0	0	3,497	3,497
09/13/20	10	10	6,345	6,345	96	96	4,408	4,408	0	0	3,330	3,330
09/14/20	8	8	6,396	6,174	179	179	3,335	3,335	27,404	27,404	2,621	2,621
09/15/20	12	12	7,826	7,826	145	145	4,771	4,771	9,437	9,437	3,109	3,109
09/16/20	9	9	10,191	10,267	143	143	4,444	4,444	11,193	11,193	3,991	3,991
09/17/20	32	32	10,239	10,239	114	114	3,182	3,182	11,291	11,291	3,400	3,400
09/18/20	14	14	13,233	13,221	214	214	4,841	4,841	14,389	14,389	4,322	4,322
09/19/20	10	10	13,560	13,400	423	423	5,167	5,167	0	0	4,422	4,422
09/20/20	12	12	10,536	10,536	270	270	3,542	3,542	0	0	3,899	3,899
09/21/20	6	6	5,539	5,539	181	181	2,917	2,917	31,428	31,428	4,376	4,376
09/22/20	10	10	10,084	9,961	244	244	4,683	4,683	10,799	10,799	4,926	4,926
09/23/20	7	7	13,291	13,156	167	167	4,786	4,786	11,289	11,289	6,180	6,180
09/24/20	8	8	15,850	15,905	185	185	5,408	5,408	10,653	10,653	6,635	6,635
09/25/20	15	15	16,104	15,994	154	154	5,401	5,401	12,272	12,272	6,874	6,874
09/26/20	14	14	14,220	14,219	146	146	5,573	5,573	0	0	6,041	6,041
09/27/20	21	21	10,635	10,635	166	166	3,886	3,886	0	0	5,692	5,692
09/28/20	12	12	4,299	4,299	247	247	3,400	3,400	31,785	31,785	4,052	4,052
09/29/20	19	19	8,005	7,856	240	240	4,446	4,446	0	0	7,143	7,143
09/30/20	9	9	14,366	14,242	112	112	5,053	5,053	20,922	20,922	7,109	7,109
10/01/20	12	12	13,051	13,025	158	158	5,099	5,099	9,419	9,419	6,914	6,914
10/02/20	10	10	12,436	12,324	204	204	4,775	4,775	11,325	11,325	6,980	6,980
10/03/20	16	16	16,488	16,514	171	171	4,863	4,863	0	0	12,872	12,872
10/04/20	20	20	12,504	12,504	146	146	3,712	3,712	0	0	22,961	22,961
10/05/20	12	12	5,639	5,375	157	157	4,478	28,115	23,480	23,480	12,601	12,601
10/06/20	7	7	11,310	11,310	117	117	4,828	4,828	11,998	11,998	14,551	14,551
10/07/20	9	9	18,953	18,823	178	178	4,580	4,580	10,491	10,491	14,165	14,165
10/08/20	23	23	17,402	17,340	182	182	5,300	5,300	12,423	12,423	17,541	17,541
10/09/20	15	15	21,088	20,697	96	96	5,532	5,532	12,788	12,788	13,864	13,864
10/10/20	21	21	26,677	26,677	163	163	4,308	4,308	0	0	15,165	15,165
10/11/20	27	27	15,937	15,994	100	100	3,175	3,175	0	0	12,872	12,872

10/12/20	7	7	9,212	8,715	63	63	3,542	3,542	27,856	27,856	14,010	14,010
10/13/20	20	20	13,626	13,626	81	81	4,295	4,295	7,118	7,118	17,236	17,236
10/14/20	11	11	23,002	22,643	124	124	4,056	4,056	11,970	11,970	19,728	19,728
10/15/20	24	24	29,757	29,757	48	48	5,514	5,514	13,318	13,318	18,981	18,981
10/16/20	13	13	25,632	25,410	217	217	6,751	6,751	15,186	15,186	15,635	15,635
10/17/20	13	13	32,104	32,139	121	121	5,447	5,447	0	0	16,171	16,171
10/18/20	13	13	29,837	29,837	135	135	4,119	4,119	0	0	16,981	16,981
10/19/20	19	19	14,289	13,538	101	101	3,699	3,699	37,889	37,889	18,822	18,822
10/20/20	11	11	20,376	20,376	97	97	5,788	5,788	13,873	13,873	21,331	21,331
10/21/20	14	14	27,422	26,797	145	145	6,845	6,845	16,973	16,973	26,694	26,694
10/22/20	18	18	40,813	40,813	108	108	6,612	6,612	20,986	20,986	21,247	21,247
10/23/20	31	31	42,837	42,213	122	122	6,604	6,604	19,851	19,851	20,542	20,542
10/24/20	15	15	45,265	45,265	134	134	6,025	6,025	0	0	23,012	23,012
10/25/20	17	17	6,616	6,554	146	146	4,360	4,360	0	0	19,790	19,790
10/26/20	16	16	73,010	72,438	114	114	4,166	4,166	52,188	52,188	20,891	20,891
10/27/20	42	42	34,482	34,458	64	64	5,942	5,942	18,418	18,418	22,911	22,911
10/28/20	47	47	37,550	36,719	146	146	5,595	5,595	19,765	19,765	24,711	24,711
10/29/20	25	25	46,612	46,636	434	434	5,948	5,948	23,580	23,580	23,068	23,068
10/30/20	33	33	49,474	49,473	209	209	6,000	6,000	25,595	25,595	24,410	24,410
10/31/20	24	24	35,070	35,101	85	85	6,151	6,151	0	0	21,915	21,915
11/01/20	24	24	46,179	46,179	248	248	4,430	4,430	0	0	23,254	23,254
11/02/20	49	49	106,091	104,707	228	228	3,763	3,763	55,019	55,019	18,954	18,954
11/03/20	17	17	72,601	72,298	128	128	5,250	5,250	18,669	18,669	20,052	20,052
11/04/20	28	28	56,225		252	252	5,225	5,225	25,042	25,042	25,191	25,191
11/05/20	36	36	57,352	57,352	217	217	5,567	5,567	21,908	21,908	24,150	24,150
11/06/20	33	33	60,711	60,711	284	284	5,931	5,931	22,516	22,516	23,303	23,303
11/07/20	28	28	86,655	86,655	214	214	6,810	6,810	0	0	24,957	24,957
11/08/20	33	33	38,562	38,562	337	337	5,887	5,887	0	0	20,572	20,572
11/09/20	22	22	20,739	20,739	171	171	4,960	4,960	52,386	52,386	21,380	21,380
11/10/20	17	17	22,322	22,322	130	130	5,746	5,746	0	0	20,433	20,433
11/11/20	15	15	36,061	36,061	289	289	7,646	7,646	36,491	36,491	22,964	22,964
11/12/20	8	8	32,388	32,388	224	224	5,658	5,658	19,511	19,511	33,487	33,487
11/13/20	18	18	23,914	23,914	185	185	5,558	5,558	21,371	21,371	27,301	27,301
11/14/20	13	13	31,957	31,957	387	387	5,860	5,860	0	0	26,860	26,860
11/15/20	8	8	27,192	27,192	231	231	3,269	3,269	0	0	24,962	24,962
11/16/20	14	14	9,635	9,635	237	237	2,874	2,874	38,273	38,273	21,406	21,406
11/17/20	9	9	45,361	45,361	306	306	1,757	1,757	13,159	13,159	20,051	20,051
11/18/20	12	12	28,619	28,619	342	342	3,918	3,918	15,318	15,318	19,631	19,631
11/19/20	17	17	20,560	20,560	243	243	4,472	4,472	16,233	16,233	22,926	22,926
11/20/20	16	16	23,102	23,102	283	283	6,426	6,426	15,156	15,156	20,253	20,253
11/21/20	17	17	17,668	17,668	236	236	6,719	6,719	0	0	19,889	19,889
11/22/20	11	11	13,130	13,130	301	301	9,187	9,187	0	0	18,662	18,662
11/23/20	22	22	4,754	4,754	197	197	7,483	7,483	25,886	25,886	15,450	15,450
11/24/20	5	5	9,690	9,690	241	241	10,794	10,794	12,228	12,228	11,329	11,329
11/25/20	21	21	16,488	16,488	484	484	10,335	10,335	10,222	10,222	18,236	18,236
11/26/20	5	5	12,901	12,901	225	225	8,107	8,107	12,289	12,289	17,555	17,555
11/27/20	6	6	12,561	12,561	473	473	12,081	12,081	10,853	10,853	14,810	14,810
11/28/20	11	11	12,477	12,477	356	356	10,728	10,728	0	0	15,871	15,871
11/29/20	18	18	9,763	9,763	201	201	5,668	5,668	0	0	12,155	12,155
11/30/20	12	12	4,030	4,030	314	314	6,472	6,472	19,979	19,979	12,411	12,411
12/01/20	9	9	8,256	8,256	576	576	8,819	8,819	8,257	8,257	13,471	13,471
12/02/20	17	17	14,071	14,071	374	374	11,251	11,251	9,331	9,331	16,226	16,226
12/03/20	18	18	12,605	12,605	203	203	11,030	11,030	10,127	10,127	14,939	14,939

12/04/20	15	15	11,300	11,300	286	286	12,127	12,127	8,745	8,745	16,366	16,366
12/05/20	18	18	12,882	12,882	290	290	11,625	11,625	0	0	15,562	15,562
12/06/20	15	15	10,949	10,949	311	311	7,455	7,455	0	0	17,367	17,367
12/07/20	15	15	3,411	3,411	701	701	6,399	6,399	17,681	17,681	14,749	14,749
12/08/20	12	12	13,819	13,819	0	0	11,006	11,006	0	0	12,353	12,353
12/09/20	12	12	14,595	14,595	1,859	1,859	11,974	11,974	9,773	9,773	16,616	16,616
12/10/20	15	15	13,750	13,750	671	671	11,897	11,897	7,955	7,955	21,022	21,022
12/11/20	13	13	13,406	13,406	639	639	12,253	12,253	10,519	10,519	21,754	21,754
12/12/20	24	24	13,947	13,947	702	702	12,057	12,057	0	0	21,552	21,552
12/13/20	16	16	11,533	11,533	461	461	8,608	8,608	0	0	18,538	18,538
12/14/20	17	17	3,063	3,063	234	234	5,930	5,930	21,309	21,309	20,327	20,327
12/15/20	12	12	11,601	11,601	402	402	11,228	11,228	10,328	10,328	18,510	18,510
12/16/20	7	7	17,615	17,615	565	565	10,297	10,297	11,078	11,078	25,261	25,261
12/17/20	12	12	18,254	18,254	628	628	11,799	11,799	12,131	12,131	35,480	35,480
12/18/20	17	17	15,811	15,811	710	710	12,248	12,248	11,815	11,815	28,507	28,507
12/19/20	23	23	17,428	17,428	631	631	12,129	12,129	0	0	27,189	27,189
12/20/20	23	23	12,858	12,858	485	485	6,870	6,870	0	0	36,031	36,031
12/21/20	15	15	5,797	5,797	197	197	5,370	5,370	22,013	22,013	33,473	33,473
12/22/20	15	15	11,795	11,795	526	526	12,511	12,511	10,654	10,654	36,841	36,841
12/23/20	17	17	14,929	14,929	489	489	11,653	11,653	12,386	12,386	39,318	39,318
12/24/20	14	14	21,634	21,634	515	515	12,485	12,485	12,662	12,662	39,059	39,059
12/25/20	20	20	20,262	20,262	446	446	9,679	9,679	0	0	32,725	32,725
12/26/20	22	22	3,093	3,093	203	203	4,974	4,974	0	0	34,693	34,693
12/27/20	21	21	8,867	8,867	248	248	6,217	6,217	0	0	32,420	32,420
12/28/20	27	27	2,960	2,960	162	162	5,996	5,996	24,462	24,462	41,414	41,414
12/29/20	24	24	11,395	11,395	308	308	12,099	12,099	14,089	14,089	53,150	53,150
12/30/20	25	25	26,457	26,457	396	396	12,406	12,406	16,716	16,716	50,043	50,043
12/31/20	19	19	19,927	19,927	539	539	12,159	12,159	18,047	18,047	55,926	55,926
01/01/21	18	18	19,358	19,358	295	295	11,091	11,091	0	0	53,286	53,286
01/02/21	28	28	3,466	3,466	201	201	6,359	6,359	0	0	57,724	57,724
01/03/21	33	33	12,489	12,489	210	210	5,211	5,211	0	0	55,052	55,052
01/04/21	33	33	4,022	4,022	128	128	6,464	6,464	30,579	30,579	58,805	58,805
01/05/21	32	32	20,733	20,733	357	357	11,271	11,271	23,700	23,700	60,938	60,938
01/06/21	63	63	25,143	25,143	295	295	13,345	13,345	0	0	62,352	62,352
01/07/21	53	53	21,703	21,703	372	372	13,734	13,734	42,360	42,360	52,634	52,634
01/08/21	33	33	19,814	19,814	222	222	14,362	14,362	25,456	25,456	68,066	68,066
01/09/21	69	69	20,177	20,177	258	258	16,105	16,105	0	0	59,974	59,974
01/10/21	103	103	15,944	15,944	188	188	10,003	10,003	0	0	54,957	54,957
01/11/21	55	55	3,717	3,717	66	66	7,594	7,594	61,422	61,422	46,191	46,191
01/12/21	115	115	19,694	19,694	140	140	14,395	14,395	25,438	25,438	45,544	45,544
01/13/21	138	138	23,852	23,852	137	137	15,873	15,873	38,869	38,869	47,539	47,539
01/14/21	144	144	21,228	21,228	0	0	16,468	16,468	35,878	35,878	48,695	48,695
01/15/21	130	130	21,271	21,271	0	0	21,366	21,366	40,197	40,197	55,777	55,777
01/16/21	109	109	21,406	21,406	0	0	20,523	20,523	0	0	41,345	41,345
01/17/21	109	109	16,642	16,642	0	0	11,170	11,170	0	0	38,605	38,605
01/18/21	118	118	3,736	3,736	0	0	8,074	8,074	84,287	84,287	37,542	37,542
01/19/21	103	103	24,023	24,023	449	449	18,894	18,894	34,291	34,291	33,371	33,371
01/20/21	144	144	26,758	26,758	94	94	20,548	20,548	41,576	41,576	38,917	38,917
01/21/21	110	110	22,587	22,587	178	178	22,339	22,339	44,357	44,357	37,901	37,901
01/22/21	100	100	23,292	23,292	129	129	21,007	21,007	42,885	42,885	40,268	40,268
01/23/21	80	80	24,093	24,093	109	109	20,057	20,057	0	0	33,568	33,568
01/24/21	124	124	18,436	18,436	105	105	10,872	10,872	0	0	30,037	30,037
01/25/21	82	82	4,240	4,240	39	39	8,521	8,521	93,822	93,822	22,218	22,218

01/26/21	75	75	22,086	22,086	73	73	17,165	17,165	36,435	36,435	20,101	20,101
01/27/21	54	54	26,916	26,916	53	53	17,944	17,944	40,285	40,285	25,334	25,334
01/28/21	52	52	23,770	23,770	110	110	18,670	18,670	34,899	34,899	28,709	28,709
01/29/21	52	52	23,205	23,205	66	66	16,374	16,374	38,118	38,118	29,079	29,079
01/30/21	92	92	24,045	24,045	43	43	15,337	15,337	0	0	23,360	23,360
01/31/21	42	42	19,336	19,336	46	46	7,030	7,030	0	0	21,140	21,140
02/01/21	30	30	4,347	4,347	27	27	5,448	5,448	79,686	79,686	18,653	18,653
02/02/21	25	25	23,337	23,337	45	45	4,384	4,384	29,064	29,064	16,879	16,879
02/03/21	30	30	26,362	26,362	34	34	12,153	12,153	31,596	31,596	19,202	19,202
02/04/21	20	20	0	0	50	50	0	0	29,960	29,960	20,674	20,674
02/05/21	12	12	45,587	45,587	54	54	26,626	26,626	28,565	28,565	19,160	19,160
02/06/21	11	11	20,586	20,586	32	32	13,209	13,209	0	0	18,285	18,285
02/07/21	14	14	19,715	19,715	27	27	6,065	6,065	0	0	15,856	15,856
02/08/21	14	14	4,317	4,317	12	12	3,868	3,868	47,095	47,095	14,126	14,126
02/09/21	14	14	19,004	19,004	23	23	10,738	10,738	16,402	16,402	12,385	12,385
02/10/21	2	2	25,387	25,387	28	28	11,138	11,138	18,114	18,114	13,024	13,024
02/11/21	12	12	21,063	21,063	31	31	10,677	10,677	17,853	17,853	13,505	13,505
02/12/21	8	8	20,701	20,701	37	37	10,388	10,388	14,581	14,581	15,163	15,163
02/13/21	7	7	21,231	21,231	40	40	9,741	9,741	0	0	13,316	13,316
02/14/21	9	9	16,675	16,675	0	0	4,099	4,099	0	0	10,982	10,982
02/15/21	16	16	4,376	4,376	36	36	3,098	3,098	30,251	30,251	9,770	9,770
02/16/21	7	7	19,590	19,590	8	8	8,683	8,683	10,057	10,057	10,630	10,630
02/17/21	11	11	25,018	25,018	39	39	8,988	8,988	10,829	10,829	12,721	12,721
02/18/21	10	10	22,501	22,501	52	52	9,099	9,099	14,515	14,515	12,060	12,060
02/19/21	8	8	24,116	24,116	14	14	7,829	7,829	11,435	11,435	12,034	12,034
02/20/21	7	7	22,371	22,371	31	31	7,785	7,785	0	0	10,408	10,408
02/21/21	11	11	22,046	22,046	14	14	3,104	3,104	0	0	9,835	9,835
02/22/21	10	10	4,646	4,646	8	8	2,252	2,252	20,849	20,849	10,645	10,645
02/23/21	12	12	20,185	20,185	22	22	8,634	8,634	7,461	7,461	8,489	8,489
02/24/21	7	7	31,519	31,519	18	18	8,642	8,642	9,212	9,212	9,939	9,939
02/25/21	6	6	25,403	25,403	39	39	8,462	8,462	9,568	9,568	9,986	9,986
02/26/21	10	10	25,207	25,207	22	22	7,512	7,512	8,341	8,341	8,524	8,524
02/27/21	6	6	24,110	24,110	13	13	7,246	7,246	0	0	7,435	7,435
02/28/21	19	19	19,952	19,952	22	22	2,810	2,810	0	0	6,039	6,039
03/01/21	11	11	4,703	4,703	10	10	2,343	2,343	15,978	15,978	5,455	5,455
03/02/21	10	10	22,857	22,857	28	28	7,913	7,913	7,223	-74,347	6,392	6,392
03/03/21	10	10	26,788	26,788	13	13	7,793	7,793	6,137	6,137	6,386	6,386
03/04/21	9	9	25,279	25,279	18	18	7,521	7,521	6,037	6,037	6,574	6,574
03/05/21	10	10	23,507	23,507	26	26	6,797	6,797	6,654	6,654	5,946	5,946
03/06/21	13	13	23,392	23,392	0	0	6,561	6,561	0	0	6,039	6,039
03/07/21	19	19	21,825	21,825	12	12	2,734	2,734	0	0	5,177	5,177
03/08/21	8	8	5,502	5,502	15	15	1,877	1,877	11,958	11,958	4,713	4,713
03/09/21	5	5	23,127	23,127	11	11	7,407	7,407	4,012	4,012	5,767	5,767
03/10/21	11	11	29,627	29,627	30	30	6,602	6,602	7,119	7,119	5,926	5,926
03/11/21	9	9	28,033	28,033	15	15	6,469	6,469	6,255	6,255	6,753	6,753
03/12/21	7	7	25,038	25,038	9	9	6,816	6,816	5,348	5,348	6,609	6,609
03/13/21	10	10	29,759	29,759	0	0	6,104	6,104	0	0	5,534	5,534
03/14/21	5	5	26,428	26,428	37	37	2,415	2,415	0	0	4,618	4,618
03/15/21	13	13	6,444	6,444	10	10	1,439	1,439	11,358	11,358	5,090	5,090
03/16/21	4	4	30,002	30,002	2	2	1,278	1,278	4,962	4,962	5,295	5,295
03/17/21	6	6	38,468	38,468	14	14	6,455	6,455	6,092	6,092	5,758	5,758
03/18/21	11	11	35,031	35,031	18	18	6,726	6,726	6,216	6,216	6,303	6,303
03/19/21	4	4	35,088	35,088	26	26	5,722	5,722	0	0	4,802	4,802

03/20/21	12	12	35,327	35,327	0	0	5,729	5,729	0	0	5,587	5,587
03/21/21	7	7	30,653	30,653	20	20	2,133	2,133	0	0	5,312	5,312
03/22/21	9	9	15,792	15,792	16	16	1,388	1,388	16,471	16,471	5,342	5,342
03/23/21	10	10	14,678	14,678	32	32	5,881	5,881	5,516	5,516	5,380	5,380
03/24/21	11	11	65,373	65,373	0	0	5,714	5,714	0	0	5,604	5,604
03/25/21	11	11	45,641	45,641	15	15	5,787	5,787	13,419	13,419	6,220	6,220
03/26/21	12	12	41,869	41,869	17	17	5,303	5,303	7,586	7,586	6,187	6,187
03/27/21	11	11	42,619	42,619	16	16	5,059	5,059	0	0	3,865	3,865
03/28/21	12	12	37,119	37,119	27	27	1,646	1,646	0	0	3,862	3,862
03/29/21	8	8	9,094	9,094	26	26	1,293	1,293	15,501	15,501	4,654	4,654
03/30/21	11	11	30,702	30,702	19	19	5,067	5,067	4,994	4,994	4,043	4,043
03/31/21	16	16	59,038	59,038	28	28	5,977	5,977	8,534	8,534	4,052	4,052
04/01/21	9	9	50,659	50,659	22	22	5,381	5,381	7,041	7,041	4,479	4,479
04/02/21	26	26	46,677	46,677	35	35	3,089	3,089	0	0	3,402	3,402
04/03/21	20	20	165	165	38	38	1,838	1,838	9,571	9,571	3,423	3,423
04/04/21	33	33	80,629	80,629	0	0	1,263	1,263	0	0	2,297	2,297
04/05/21	24	24	10,793	10,793	54	54	1,247	1,247	10,360	10,360	2,762	2,762
04/06/21	12	12	8,045	8,045	7	7	4,804	4,804	6,623	6,623	2,380	2,380
04/07/21	24	24	922	922	23	23	5,370	5,370	8,788	8,788	2,762	2,762
04/08/21	21	21	97,028	97,028	16	16	5,140	5,140	9,901	9,901	3,030	3,030
04/09/21	14	14	153	153	24	24	5,045	5,045	10,875	10,875	2,865	-4,860
04/10/21	10	10	1,403	1,403	27	27	6,356	6,356	0	0	2,584	2,584
04/11/21	16	16	117,900	117,900	27	27	1,793	1,793	0	0	1,730	1,730
04/12/21	9	9	8,536	8,536	34	34	1,627	1,627	22,744	22,744	3,568	3,568
04/13/21	12	12	39,113	39,113	30	30	4,512	4,512	6,292	6,292	2,473	2,473
04/14/21	10	10	43,505	43,505	33	33	4,894	4,894	10,474	10,474	2,491	2,491
04/15/21	11	11	38,045	38,045	26	26	4,189	4,189	9,663	9,663	2,671	2,671
04/16/21	15	15	36,442	36,442	47	47	4,504	4,504	10,598	10,598	2,756	2,756
04/17/21	16	16	35,861	35,861	30	30	4,157	4,157	0	0	2,206	2,206
04/18/21	11	11	29,377	29,377	38	38	1,506	1,506	0	0	1,882	1,882
04/19/21	10	10	6,696	6,696	18	18	1,308	1,308	21,071	21,071	2,963	2,963
04/20/21	21	21	44,063	44,063	26	26	4,262	4,262	0	0	2,524	2,524
04/21/21	6	6	34,921	34,921	53	53	4,639	4,639	17,718	17,718	2,398	2,398
04/22/21	19	19	33,400	33,400	54	54	3,708	3,708	10,814	10,814	2,728	2,728
04/23/21	9	9	32,340	32,340	46	46	3,911	3,911	11,731	11,731	2,734	2,734
04/24/21	13	13	32,633	32,633	0	0	3,308	3,308	0	0	2,061	2,061
04/25/21	11	11	24,505	24,505	80	80	1,653	1,653	0	0	1,712	1,712
04/26/21	11	11	5,952	5,952	60	60	1,143	1,143	19,852	19,852	2,064	2,064
04/27/21	12	12	30,317	30,317	22	22	3,592	3,592	7,665	7,665	2,685	2,685
04/28/21	20	20	31,539	31,539	29	29	3,818	3,818	8,665	8,665	2,110	2,110
04/29/21	13	13	26,538	26,538	31	31	3,990	3,990	10,143	10,143	2,445	2,445
04/30/21	16	16	24,299	24,299	69	69	3,821	3,821	9,135	9,135	2,381	2,381
05/01/21	15	15	25,670	25,670	39	39	3,025	3,025	0	0	1,907	1,907
05/02/21	11	11	9,921	9,921	0	0	1,093	1,093	0	0	1,671	1,671
05/03/21	17	17	3,760	3,760	68	68	1,027	1,027	16,353	16,353	1,649	1,649
05/04/21	7	7	24,371	24,371	59	59	3,064	3,064	4,515	4,515	1,947	1,947
05/05/21	5	5	26,000	26,000	70	70	3,021	3,021	6,317	6,317	2,144	2,144
05/06/21	13	13	21,712	21,712	50	50	2,846	2,846	7,960	7,960	2,613	2,613
05/07/21	7	7	19,124	19,124	72	72	3,043	3,043	8,186	8,186	2,490	2,490
05/08/21	12	12	20,779	20,779	84	84	2,743	2,743	0	0	2,047	2,047
05/09/21	11	11	9,128	9,128	47	47	1,175	1,175	0	0	1,770	1,770
05/10/21	14	14	3,292	3,292	29	29	704	704	13,984	13,984	2,358	2,358
05/11/21	16	16	19,791	19,791	43	43	1,897	1,897	4,941	4,941	2,474	2,474

05/12/21	9	9	21,498	21,498	60	60	3,090	3,090	6,418	6,418	2,284	2,284
05/13/21	7	7	19,461	19,461	91	91	3,632	3,632	5,701	5,701	2,656	2,656
05/14/21	14	14	7,025	7,025	96	96	2,880	2,880	6,347	6,347	2,193	2,193
05/15/21	18	18	15,685	15,685	0	0	2,695	2,695	0	0	2,027	2,027
05/16/21	25	25	14,026	14,026	105	105	1,233	1,233	0	0	1,926	1,926
05/17/21	22	22	3,350	3,350	80	80	822	822	11,061	11,061	1,981	1,981
05/18/21	14	14	17,210	17,210	71	71	2,767	2,767	3,988	3,988	2,220	-2,364
05/19/21	12	12	19,050	19,050	164	164	2,000	2,000	6,080	6,080	2,135	2,135
05/20/21	24	24			129	129	2,628	2,628	5,733	5,733	2,694	2,694
05/21/21	10	10	12,800	12,800	147	147	2,604	2,604	4,792	4,792	2,702	2,702
05/22/21	19	19	12,611	12,611	137	137	2,586	2,586	0	0	2,523	2,523
05/23/21	18	18	9,766	9,766	227	227	1,274	1,274	0	0	2,092	2,092
05/24/21	15	15	2,229	2,229	185	185	703	703	11,067	11,067	2,368	2,368
05/25/21	13	13	3,155	3,155	155	155	2,483	2,483	5,359	5,359	2,410	2,410
05/26/21	19	19	12,646	12,646	207	207	2,932	2,932	5,007	5,007	2,987	2,987
05/27/21	7	7	13,933	13,933	313	313	3,050	3,050	5,290	5,290	3,380	3,380
05/28/21	16	16	11,268	11,268	637	637	3,006	3,006	5,482	5,482	4,028	4,028
05/29/21	11	11	11,526	11,526	700	700	2,725	2,725	0	0	3,240	3,240
05/30/21	27	27	8,662	8,662	692	692	1,307	1,307	0	0	3,111	3,111
05/31/21	23	23	360	360	524	524	932	932	9,732	9,732	3,284	3,284
06/01/21	24	24	9,848	9,848	614	614	6,917	6,917	4,388	4,388	3,099	3,099
06/02/21	24	24	1,350	1,350	915	915	3,269	3,269	4,984	4,984	4,261	4,261
06/03/21	24	24	15,554	15,554	1,083	1,083	2,894	2,894	5,250	5,250	5,179	5,179
06/04/21	24	24	6,953	6,953	1,247	1,247	2,809	2,809	4,969	4,969	6,140	6,140
06/05/21	30	30	6,654	6,654	670	670	2,071	2,071	0	0	5,651	5,651
06/06/21	19	19	5,180	5,180	1,259	1,259	1,979	1,979	0	0	5,223	5,223
06/07/21	33	33	1,164	1,164	1,026	1,026	881	881	9,542	9,542	5,594	5,594
06/08/21	16	16	6,018	6,018	708	708	3,449	3,449	3,504	3,504	5,966	5,966
06/09/21	21	21	5,557	5,557	842	842	3,855	3,855	4,427	4,427	7,319	7,319
06/10/21	22	22	4,475	4,475	1,438	1,438	3,672	3,672	14,004	14,004	7,237	7,237
06/11/21	35	35	3,871	3,871	1,566	1,566	3,282	3,282	4,142	4,142	7,958	7,958
06/12/21	34	34	3,972	3,972	1,735	1,735	3,649	3,649	0	0	7,550	7,550
06/13/21	23	23	2,973	2,973	1,727	1,727	1,707	1,707	0	0	7,330	7,330
06/14/21	20	20	689	689	1,122	1,122	1,175	1,175	8,167	8,167	7,619	7,619
06/15/21	21	21	3,235	3,235	1,422	1,422	4,250	4,250	3,432	3,432	7,587	7,587
06/16/21	19	19	3,058	3,058	1,110	1,110	3,789	3,789	3,832	3,832	8,808	8,808
06/17/21	23	23	2,044	2,044	1,584	1,584	4,253	4,253	4,197	4,197	10,821	10,821
06/18/21	30	30	3,181	3,181	1,564	1,564	4,098	4,098	4,214	4,214	10,272	10,272
06/19/21	23	23	2,624	2,624	1,397	1,397	0	0	0	0	10,075	10,075
06/20/21	17	17	1,909	1,909	1,367	1,367	5,542	5,542	0	0	9,074	9,074
06/21/21	25	25	487	487	1,136	1,136	1,268	1,268	7,209	7,209	10,467	10,467
06/22/21	24	24	2,204	2,204	722	722	4,233	4,233	4,040	4,040	11,503	11,503
06/23/21	16	16	2,320	2,320	859	859	4,963	4,963	4,341	4,341	15,882	15,882
06/24/21	24	24	2,007	2,007	1,277	1,277	5,340	5,340	4,507	4,507	16,702	16,702
06/25/21	25	25	1,986	1,986	1,025	1,025	5,270	5,270	4,924	4,924	15,364	15,364
06/26/21	14	14	2,128	2,128	943	943	5,051	5,051	0	0	17,944	17,944
06/27/21	22	22	1,634	1,634	889	889	2,384	2,384	0	0	14,624	14,624
06/28/21	17	17	509	509	695	695	1,661	1,661	10,179	10,179	22,717	22,717
06/29/21	9	9	3,395	3,395	345	345	5,711	5,711	7,091	7,091	20,252	20,252
06/30/21	12	12	1,376	1,376	543	543	6,105	6,105	9,227	9,227	25,667	25,667
07/01/21	18	18	2,664	2,664	1,057	1,057	6,081	6,081	12,345	12,345	27,621	27,621
07/02/21	23	23	2,683	2,683	1,048	1,048	5,842	5,842	12,563	12,563	26,773	26,773
07/03/21	14	14	3,006	3,006	770	770	6,265	6,265	0	0	24,447	24,447

07/04/21	22	22	2,605	2,605	784	784	2,611	2,611	0	0	23,824	23,824
07/05/21	23	23	796	796	480	480	1,805	1,805	32,607	32,607	27,100	27,100
07/06/21	57	57	3,585	3,585	438	438	7,989	7,989	14,137	14,137	28,611	28,611
07/07/21	17	17	4,081	4,081	425	425	8,507	8,507	17,384	17,384	32,048	32,048
07/08/21	23	23	4,442	4,442	602	602	9,452	9,452	17,317	17,317	32,080	32,080
07/09/21	26	26	4,580	4,580	559	559	9,319	9,319	21,879	21,879	35,504	35,504
07/10/21	0	0	4,696	4,696	615	615	9,581	9,581	0	0	31,800	31,800
07/11/21	51	51	4,314	4,314	522	522	3,779	3,779	0	0	31,352	31,352
07/12/21	29	29	1,260	1,260	479	479	3,074	3,074	33,932	33,932	34,455	34,455
07/13/21	24	24	6,950	6,950	438	438	11,137	11,137	43,960	43,960	36,443	36,443
07/14/21	28	28	1,450	1,450	480	480	12,116	12,116	26,390	26,390	41,960	41,960
07/15/21	36	36	11,042	11,042	406	406	12,821	12,821	27,688	27,688	48,173	48,173
07/16/21	30	30	10,908	10,908	434	434	12,420	12,420	31,060	31,060	51,485	51,485
07/17/21	33	33	11,005	11,005	460	460	12,631	12,631	0	0	53,980	53,980
07/18/21	31	31	12,532	12,532	417	417	4,438	4,438	0	0	47,599	47,599
07/19/21	65	65	4,151	4,151	265	265	5,307	5,307	61,628	61,628	40,394	40,394
07/20/21	0	0	18,181	18,181	254	254	13,853	13,853	27,286	27,286	46,429	46,429
07/21/21	72	72	21,539	21,539	252	252	15,198	15,198	30,587	30,587	43,623	43,623
07/22/21	48	48	22,061	22,061	193	193	16,244	16,244	29,535	29,535	39,570	39,570
07/23/21	72	72	19,409	19,409	355	355	0	0	31,171	31,171	35,841	35,841
07/24/21	46	-5	25,688	25,688	405	405	32,244	32,244	0	0	31,291	31,291
07/25/21	76	76	15,242	15,242	375	375	6,535	6,535	0	0	28,656	28,656
07/26/21	71	71	5,307	5,307	234	234	5,920	5,920	61,625	61,625	24,741	24,741
07/27/21	86	86	27,024	27,024	71	71	17,408	17,408	26,399	26,399	23,362	23,362
07/28/21	49	49	27,781	27,781	327	327	19,028	19,028	27,149	27,149	25,512	25,512
07/29/21	66	66	25,190	25,190	160	160	19,223	19,223	26,689	26,689	30,724	30,724
07/30/21	53	53	24,309	24,309	393	393	19,346	19,346	24,753	24,753	29,294	29,294
07/31/21	77	77	23,689	23,689	252	252	18,809	18,809	0	0	25,760	25,760
08/01/21	96	96	19,714	19,714	268	268	6,740	6,740	0	0	24,141	24,141
08/02/21	90	90	8,160	8,160	230	230	6,506	6,506	55,939	55,939	21,687	21,687
08/03/21	96	96	23,919	23,919	112	112	18,911	18,911	20,327	20,327	21,693	21,693
08/04/21	85	85	28,784	28,784	202	202	20,685	20,685	21,874	21,874	29,000	29,000
08/05/21	124	124	26,460	26,460	165	165	21,569	21,569	21,387	21,387	29,873	29,873
08/06/21	107	107	25,077	25,077	322	322	21,563	21,563	21,561	21,561	31,482	31,482
08/07/21	96	96	25,857	25,857	248	248	20,018	20,018	0	0	28,233	28,233
08/08/21	125	125	20,460	20,460	249	249	7,573	7,573	0	0	27,117	27,117
08/09/21	143	143	5,775	5,775	152	152	6,513	6,513	39,638	39,638	24,962	24,962
08/10/21	111	111	27,815	27,815	80	80	19,555	19,555	15,680	15,680	23,339	23,339
08/11/21	81	81	31,852	31,852	112	112	22,711	22,711	17,023	17,023	29,289	29,289
08/12/21	99	99	28,554	28,554	157	157	24,975	24,975	17,410	17,410	32,763	32,763
08/13/21	66	66	26,453	26,453	273	273	22,758	22,758	15,657	15,657	32,426	32,426
08/14/21	53	53	24,427	24,427	181	181	23,642	23,642	0	0	29,153	29,153
08/15/21	51	51	21,269	21,269	309	309	0	0	0	0	26,440	26,440
08/16/21	42	42	11,186	11,186	137	137	16,467	16,467	25,726	25,726	28,278	28,278
08/17/21	28	28	22,959	22,959	62	62	14,814	14,814	14,336	14,336	26,628	26,628
08/18/21	46	46	28,405	28,405	108	108	28,953	28,953	11,956	11,956	33,646	33,646
08/19/21	33	33	23,973	23,973	159	159	23,169	23,169	12,445	12,445	36,421	36,421
08/20/21	20	20	22,319	22,319	0	0	21,734	21,734	12,450	12,450	37,038	37,038
08/21/21	32	32	22,712	22,712	0	0	20,307	20,307	0	0	31,783	31,783
08/22/21	21	21	17,300	17,300	0	0	7,658	7,658	0	0	31,976	31,976
08/23/21	35	35	5,166	5,166	67	21,324	6,543	6,543	23,899	23,899	31,798	31,798
08/24/21	20	20	24,853	24,853	123	123	18,262	18,262	10,072	10,072	30,653	30,653
08/25/21	26	26	23,706	23,706	137	137	21,250	21,250	10,781	10,781	35,584	35,584

08/26/21	32	32	19,683	19,683	104	104	20,633	20,633	7,115	7,115	38,002	38,002
08/27/21	21	21	457	457	186	186	19,556	19,556	9,489	9,489	37,738	37,738
08/28/21	33	33	35,630	35,630	217	217	17,546	17,546	0	0	32,087	32,087
08/29/21	23	23	13,630	13,630	188	188	6,837	6,837	0	0	32,937	32,937
08/30/21	37	37	7,688	7,688	93	93	5,564	5,564	15,489	15,489	26,227	26,227
08/31/21	19	19	24	24	90	90	11,146	11,146	7,767	7,767	32,053	32,053
09/01/21	28	28	33,129	33,129	160	160	17,337	17,337	6,818	6,818	35,534	35,534
09/02/21	28	28	14,153	14,153	133	133	18,138	18,138	9,561	9,561	37,870	37,870
09/03/21	28	28	15,224	15,224	169	169	17,409	17,409	6,311	6,311	42,114	42,114
09/04/21	28	28	13,336	13,336	89	89	15,586	15,586	0	0	36,642	36,642
09/05/21	18	18	10,410	10,410	115	115	7,504	7,504	0	0	36,515	36,515
09/06/21	36	36	3,050	3,050	81	81	5,127	5,127	9,357	9,357	40,938	40,938
09/07/21	19	19	14,534	14,534	52	52	15,784	15,784	5,528	5,528	37,225	37,225
09/08/21	28	28	5,922	5,922	133	133	15,876	15,876	5,618	5,618	38,510	38,510
09/09/21	17	17	18,070	18,070	143	143	14,828	14,828	4,763	4,763	37,521	37,521
09/10/21	25	25	9,966	9,966	176	176	0	0	4,440	4,440	36,777	36,777
09/11/21	46	46	9,601	9,601	130	130	26,744	26,744	0	0	28,856	28,856
09/12/21	49	49	7,679	7,679	214	214	5,139	5,139	0	0	28,614	28,614
09/13/21	92	92	2,062	2,062	77	77	4,161	4,161	7,804	7,804	30,385	30,385
09/14/21	73	73	14,065	14,065	30	30	12,929	12,929	3,261	3,261	26,288	26,288
09/15/21	80	80	383	383	70	70	13,217	13,217	3,723	3,723	29,879	29,879
09/16/21	84	84	13,272	13,272	97	97	7,040	7,040	4,075	4,075	26,375	26,375
09/17/21	46	46	7,373	7,373	200	200	3,754	3,754	3,222	3,222	32,338	32,338
09/18/21	66	66	7,414	7,414	99	99	0	0	0	0	29,438	29,438
09/19/21	49	49	5,814	5,814	129	129	16,694	16,694	0	0	29,007	29,007
09/20/21	72	72	1,475	1,475	65	65	3,367	3,367	5,988	5,988	35,768	35,768
09/21/21	41	41	7,983	7,983	128	128	12,521	12,521	2,450	2,450	31,128	31,128
09/22/21	43	43	6,794	6,794	97	97	11,603	11,603	2,840	2,840	33,585	33,585
09/23/21	54	54	6,229	6,229	89	89	11,808	11,808	3,031	3,031	35,798	35,798
09/24/21	38	38	6,675	6,675	312	312	10,139	10,139	2,746	2,746	35,659	35,659
09/25/21	29	29	5,216	5,216	163	163	0	0	0	0	29,746	29,746
09/26/21	35	35	4,706	4,706	115	115	13,685	13,685	0	0	32,997	32,997
09/27/21	31	31	1,309	1,309	64	64	3,007	3,007	5,039	5,039	37,510	37,510
09/28/21	25	25	6,765	6,765	95	95	9,792	9,792	2,290	2,290	34,604	34,604
09/29/21	22	22	5,900	5,900	105	105	9,796	9,796	2,761	2,761	35,089	35,089
09/30/21	34	34	5,204	5,204	127	127	8,828	8,828	2,400	2,400	35,773	35,773
10/01/21	41	41	4,935	4,935	170	170	7,388	7,388	2,037	2,037	34,610	34,610
10/02/21	28	28	4,948	4,948	115	115	7,369	7,369	0	0	29,389	29,389
10/03/21	27	27	3,744	3,744	119	119	0	0	0	0	29,666	29,666
10/04/21	26	26	1,138	1,138	63	63	5,262	5,262	4,271	4,271	34,306	34,306
10/05/21	26	26	5,582	5,582	59	59	0	0	1,801	1,801	33,079	33,079
10/06/21	25	25	4,570	4,570	92	92	7,682	7,682	2,303	2,303	38,713	38,713
10/07/21	22	22	5,006	5,006	64	64	15,310	15,310	1,807	1,807	39,767	39,767
10/08/21	17	17	4,470	4,470	183	183	7,158	7,158	2,309	2,309	34,941	34,941
10/09/21	24	24	4,734	4,734	107	107	0	0	0	0	39,413	39,413
10/10/21	25	25	3,991	3,991	102	102	8,843	8,843	0	0	33,593	33,593
10/11/21	12	12	1,120	1,120	90	90	2,007	2,007	3,829	3,829	39,588	39,588
10/12/21	22	22	5,880	5,880	72	72	7,187	7,187	0	0	37,717	37,717
10/13/21	26	26	1,359	1,359	56	56	6,320	6,320	2,758	2,758	41,501	41,501
10/14/21	5	5	9,474	9,474	60	60	5,825	5,825	1,932	1,932	44,619	44,619
10/15/21	14	14	6,099	6,099	125	125	5,286	5,286	2,248	2,248	44,251	44,251
10/16/21	21	21	4,899	4,899	45	45	5,203	5,203	0	0	42,818	42,818
10/17/21	27	27	3,778	3,778	92	92	1,993	1,993	0	0	44,696	44,696

10/18/21	21	21	1,057	1,057	75	75	1,413	1,413	4,492	4,492	48,855	48,855
10/19/21	30	30	5,945	5,945	22	22	4,220	4,220	1,889	1,889	43,411	43,411
10/20/21	21	21	6,036	6,036	81	81	5,069	5,069	2,528	2,528	48,599	48,599
10/21/21	48	48	6,127	6,127	62	62	4,798	4,798	1,881	1,881	51,548	51,548
10/22/21	45	45	6,366	6,366	111	111	4,653	4,653	2,556	2,556	48,791	48,791
10/23/21	44	44	6,291	6,291	108	108	4,452	4,452	0	0	44,985	44,985
10/24/21	38	38	5,005	5,005	65	65	0	0	0	0	38,740	38,740
10/25/21	63	63	1,295	1,295	48	48	2,787	2,787	4,485	4,485	36,283	36,283
10/26/21	39	39	6,635	6,635	30	30	0	0	1,926	1,926	43,503	43,503
10/27/21	39	39	6,999	6,999	73	73	4,538	4,538	2,532	2,532	44,000	44,000
10/28/21	64	64	5,990	5,990	59	59	9,300	9,300	2,212	2,212	39,091	39,091
10/29/21	78	78	6,433	6,433	80	80	4,001	4,001	2,261	2,261	43,161	43,161
10/30/21	72	72	7,360	7,360	75	75	3,478	3,478	0	0	40,726	40,726
10/31/21	91	91	6,329	6,329	96	96	1,446	1,446	0	0	37,667	37,667
11/01/21	71	71	1,866	1,866	65	65	0	0	0	0	39,935	39,935
11/02/21	110	110	2,039	2,039	36	36	994	994	5,820	5,820	33,615	33,615
11/03/21	103	103	10,230	10,230	49	49	3,588	3,588	2,287	2,287	40,803	40,803
11/04/21	78	78	9,344	9,344	27	27	2,660	2,660	3,291	3,291	36,738	36,738
11/05/21	60	60	8,998	8,998	59	59	3,763	3,763	3,093	3,093	33,810	33,810
11/06/21	69	69	9,605	9,605	85	85	7,188	7,188	0	0	30,150	30,150
11/07/21	89	89	8,547	8,547	78	78	1,382	1,382	0	0	29,843	29,843
11/08/21	62	62	2,197	2,197	55	55	0	0	6,417	6,417	32,266	32,266
11/09/21	54	54	12,511	12,511	19	19	4,473	4,473	0	0	32,785	32,785
11/10/21	64	64	11,883	11,883	70	70	0	0	6,461	6,461	39,547	39,547
11/11/21	96	96	12,603	12,603	49	49	7,049	7,049	4,286	4,286	42,590	42,590
11/12/21	75	75	3,860	3,860	70	70	0	0	4,353	4,353	39,040	39,040
11/13/21	89	89	14,646	14,646	56	56	3,353	3,353	0	0	37,669	37,669
11/14/21	52	52	12,496	12,496	34	34	3,130	3,130	0	0	36,128	36,128
11/15/21	22	22	3,259	3,259	42	42	942	942	9,798	9,798	39,796	39,796
11/16/21	31	31	19,778	19,778	37	37	775	775	4,091	4,091	36,956	36,956
11/17/21	35	35	20,294	20,294	44	44	4,571	4,571	6,667	6,667	38,073	38,073
11/18/21	24	24	20,366	20,366	27	27	3,915	3,915	6,315	6,315	46,991	46,991
11/19/21	23	23	21,220	21,220	55	55	0	0	6,636	6,636	44,411	44,411
11/20/21	17	17	22,678	22,678	0	0	3,837	3,837	0	0	39,881	39,881
11/21/21	38	38	19,749	19,749	49	49	3,306	3,306	0	0	39,458	39,458
11/22/21	19	19	5,291	5,291	60	60	2,141	2,141	15,875	15,875	45,021	45,021
11/23/21	22	22	30,454	30,454	31	31	0	0	6,777	6,777	42,832	42,832
11/24/21	24	24	32,591	32,591	37	37	3,698	3,698	8,527	8,527	42,621	42,621
11/25/21	13	13	33,464	33,464	32	32	8,415	8,415	9,258	9,258	46,955	46,955
11/26/21	25	25	34,436	34,436	76	76	3,445	3,445	9,912	9,912	49,554	49,554
11/27/21	23	23	37,057	37,057	10	10	0	0	0	0	39,567	39,567
11/28/21	41	41	31,581	31,581	28	28	2,956	2,956	0	0	36,507	36,507
11/29/21	39	39	8,507	8,507	72	72	1,774	1,774	22,911	22,911	42,702	42,702
11/30/21	113	113	47,201	47,201	27	27	0	0	10,261	10,261	40,011	40,011
12/01/21	73	73	49,610	49,610	39	39	6,652	6,652	10,536	10,536	47,454	47,454
12/02/21	96	96	48,416	48,416	29	29	0	0	14,500	14,500	53,322	53,322
12/03/21	90	90	49,858	49,858	33	33	6,234	6,234	13,738	13,738	50,810	50,810
12/04/21	59	59	51,464	51,464	0	0	0	0	0	0	41,457	41,457
12/05/21	61	61	42,153	42,153	4	4	3,811	3,811	0	0	43,285	43,285
12/06/21	94	94	11,578	11,578	101	101	752	752	0	0	51,432	51,432
12/07/21	74	74	59,019	59,019	19	19	0	0	43,808	43,808	45,333	45,333
12/08/21	83	83	61,340	61,340	47	47	3,304	3,304	0	0	50,811	50,811
12/09/21	63	63	56,854	56,854	41	41	6,395	6,395	26,412	26,412	50,282	50,282

12/10/21	87	87	55,140	55,140	81	81	0	0	17,095	17,095	58,352	58,352
12/11/21	75	75	53,919	53,919	0	0	2,992	2,992	-83	-83	52,279	52,279
12/12/21	101	101	43,848	43,848	29	29	2,655	2,655	0	0	48,071	48,071
12/13/21	76	76	12,064	12,064	127	127	855	855	49,802	49,802	53,953	53,953
12/14/21	69	69	63,405	63,405	87	87	771	771	26,136	26,136	59,746	59,746
12/15/21	75	75	65,713	65,713	92	92	5,651	5,651	27,140	27,140	77,966	77,966
12/16/21	76	76	60,866	60,866	90	90	2,627	2,627	28,900	28,900	87,801	87,801
12/17/21	125	125	58,128	58,128	209	209	0	0	33,359	33,359	92,713	92,713
12/18/21	83	83	58,536	58,536	186	186	2,750	2,750	0	0	89,074	89,074
12/19/21	102	102	48,473	48,473	297	297	2,530	2,530	0	0	81,959	81,959
12/20/21	81	81	15,075	15,075	258	258	1,557	1,557	79,704	79,704	92,371	92,371
12/21/21	78	78	72,857	72,857	357	357	0	0	49,823	49,823	89,298	89,298
12/22/21	99	99	84,272	84,272	502	502	2,980	2,980	60,041	60,041	105,626	105,626
12/23/21	87	87	91,608	91,608	710	710	3,319	3,319	72,912	72,912	122,760	122,760
12/24/21	140	140	94,124	94,124	1,093	1,093	6,883	6,883	0	0	121,810	121,810
12/25/21	46	46	104,611	104,611	1,251	1,251	2,916	2,916	0	0	121,861	121,861
12/26/21	208	208	27,697	27,697	1,116	1,116	0	0	0	0	118,945	118,945
12/27/21	361	361	30,459	30,459	743	743	803	803	214,619	214,619	107,699	107,699
12/28/21	197	197	179,808	179,808	974	974	943	943	99,671	99,671	138,025	138,025
12/29/21	207	207	208,099	208,099	1,272	1,272	4,426	4,426	100,760	100,760	184,475	184,475
12/30/21	195	195	206,544	206,544	1,742	1,742	5,290	5,290	161,688	161,688	188,925	188,925
12/31/21	229	229	232,200	232,200	1,658	1,658	18,061	18,061	0	0	190,544	190,544
01/01/22	191	191	219,126	219,126	1,867	1,867	9,193	9,193	0	0	162,572	162,572
01/02/22	161	161	58,432	58,432	1,936	1,936	1,671	1,671	0	0	134,943	134,943
01/03/22	175	175	67,461	67,461	1,423	1,423	2,877	2,877	372,766	372,766	187,414	187,414
01/04/22	91	91	271,746	271,746	880	880	15,184	15,184	117,775	117,775	221,222	221,222
01/05/22	189	189	332,252	332,252	1,766	1,766	20,626	20,626	137,180	137,180	194,615	194,615
01/06/22	174	174	262,787	262,787	1,759	1,759	25,821	25,821	0	0	180,610	180,610
01/07/22	159	159	328,214	328,214	0	0	28,023	28,023	242,440	242,440	178,868	178,868
01/08/22	165	165	303,669	303,669	2,410	2,410	30,671	30,671	0	0	140,566	140,566
01/09/22	157	157	296,097	296,097	984	984	11,599	11,599	0	0	141,398	141,398
01/10/22	192	192	93,896	93,896	816	816	0	0	292,394	292,394	143,867	143,867
01/11/22	221	221	368,379	368,379	865	865	44,678	44,678	134,942	134,942	115,816	115,816
01/12/22	190	190	361,719	361,719	670	670	44,187	44,187	179,125	179,125	129,980	129,980
01/13/22	201	201	308,221	308,221	591	591	43,523	43,523	159,161	159,161	106,085	106,085
01/14/22	165	165	329,371	329,371	523	523	0	0	162,508	162,508	98,949	98,949
01/15/22	119	119	324,580	324,580	527	527	91,406	91,406			80,725	80,725
01/16/22	223	223	278,129	278,129	484	484	19,132	19,132			70,160	70,160
